# Lifespan Extension in a Semelparous Chordate Occurs via Developmental Growth Arrest Just Prior to Meiotic Entry

**DOI:** 10.1371/journal.pone.0093787

**Published:** 2014-04-02

**Authors:** Gunasekaran Subramaniam, Coen Campsteijn, Eric M. Thompson

**Affiliations:** 1 Sars International Centre for Marine Molecular Biology, University of Bergen, Bergen, Norway; 2 Department of Biology, University of Bergen, Bergen, Norway; National Cancer Institute, United States of America

## Abstract

It is proposed that the ageing process is linked to signaling from the germline such that the rate of ageing can be adjusted to the state of the reproductive system, allowing these two processes to co-evolve. Mechanistic insight into this link has been primarily derived from iteroparous reproductive models, the nematode *C. elegans*, and the arthropod *Drosophila*. Here, we examined to what extent these mechanisms are evolutionarily conserved in a semelparous chordate, *Oikopleura dioica*, where we identify a developmental growth arrest (GA) in response to crowded, diet-restricted conditions, which can extend its lifespan at least three-fold. Under nutritional stress, the iteroparative models sacrifice germ cells that have entered meiosis, while maintaining a reduced pool of active germline stem cells (GSCs). In contrast, *O. dioica* only entered GA prior to meiotic entry. Stress conditions encountered after this point led to maturation in a normal time frame but with reduced reproductive output. During GA, TOR signaling was inhibited, whereas MAPK, ERK1/2 and p38 pathways were activated, and under such conditions, activation of these pathways was shown to be critical for survival. Direct inhibition of TOR signaling alone was sufficient to prevent meiotic entry and germline differentiation. This inhibition activated the p38 pathway, but did not activate the ERK1/2 pathway. Thus, the link between reproductive status and lifespan extension in response to nutrient-limited conditions is interpreted in a significantly different manner in these iteroparative versus semelparous models. In the latter case, meiotic entry is a definitive signal that lifespan extension can no longer occur, whereas in the former, meiotic entry is not a unique chronological event, and can be largely erased during lifespan extension in response to nutrient stress, and reactivated from a pool of maintained GSCs when conditions improve.

## Introduction

The progression of the ageing process can, in a number of organisms, be paused when they encounter unfavorable environmental conditions. This allows such organisms more latitude in coupling reproductive events to circumstances favorable for survival and growth. The importance of this link is strengthened by evidence from the nematode, *Caenorhabditis elegans*, and the fruitfly, *Drosophila*, that loss of germline stem cells (GSCs) extends lifespan through an evolutionarily conserved pathway involving FOXO-family transcription factors [1]. By linking ageing to signaling from the germline, it is proposed that the rate of ageing can be adjusted to the state of the reproductive system, allowing the processes of ageing and reproduction to co-evolve [2]. The mouse, and more particularly, the *Drosophila* and *C. elegans* models have been at the centre of research on developmental growth arrest (GA), ageing/lifespan and the links of these processes to the reproductive axis. They are also used as a proxy for understanding the ageing process in humans, particularly with respect to the effects of dietary restriction. All of these models have an iteroparative mode of reproduction, with multiple reproductive cycles over the life cycle. In this study, we have focused instead on a chordate, *Oikopleura dioica*, with a semelparous mode of reproduction, in which there is only a single reproductive episode before death.

*C. elegans* can enter developmental arrest at several points in its life cycle including first stage larval “L1 arrest”, the “dauer arrest” alternative to the third larval stage, and adult reproductive diapause “ARD” [3], [4]. L1 arrest occurs in response to starvation, and larvae, which retain an L1 morphology, are able to survive for weeks. Dauers form in response to high nematode density, starvation or high temperature and exhibit a modified morphology in which a specialized cuticle is formed, the mouth is closed by an internal plug, and the pharynx is constricted and ceases pumping. The dauer can persist for months. In response to starvation, ARD results in apoptotic death within the germ line, with the exception of a small population of GSCs and allows sexually mature adults to delay reproductive onset 15-fold and expand adult lifespan 3-fold [4]. More recently, it has been questioned whether ARD is a distinct diapause state [5]and an alternative appellation “Oogenic Germline Starvation Response (OGSR)” has been proposed. Instead of a full stop in reproductive onset, this response associates germline shrinkage (as in the described ARD) with a greatly reduced rate of oogenesis and includes the capacity for germline regeneration upon resumption of feeding. The *Drosophila* germ line responds similarly to starvation; programmed cell death initiates at two points, in region 2a of the germarium where cytoblasts divide to form germline cysts which enter meiosis, and in stage 8 egg chambers at the onset of vitellogenesis [6]. As in *C. elegans* ARD/OGSR, a small population of GSCs is maintained in the *Drosophila* ovary under starvation. The *Drosophila* model has also been used to reveal the differential response of cell cycle variants to dietary restriction. Whereas mitotic cells continue to proliferate in a nutrition-independent fashion, endoreplicative cells enter a quiescent state [7].

Here we identify a developmental growth arrest in the marine zooplankton *Oikopleura dioica*, and show that lifespan can be increased at least 3-fold. Compared to the arthropod and nematode models above, *Oikopleura* is a chordate and a member of the closest extant sister group to the vertebrates [8]. Embryonic development to motility is very rapid, as is the case for many pelagic embryos [9], and in contrast to the above model organisms, the larval stage is non-feeding, with development to metamorphosis fueled by maternal reserves. The feeding mode is also different from the *C. elegans* and *Drosophila* developmental growth arrest models. *Oikopleura* begins filter-feeding after metamorphosis by inflating its first filter-feeding house, a complex gelatinous extracellular matrix [10] which completely surrounds the animal. In *O. dioica*, this house is completely re-synthesized and replaced every 4 h over the course of a short 6-day life cycle at 15°C. The rate of replacement is unaffected by available food quantity or age [11] and each house represents a substantial anabolic investment of 15% of total body carbon [12]. Similar to *Drosophila*, oogenesis occurs in a syncytial environment (coenocyst) in which germline nuclei differentiate into meiotic nuclei in prophase I and asynchronously endocycling nurse nuclei, all sharing a common cytoplasm [13]–[16]. However, unlike both *Drosophila* and *C. elegans*, there is no population of GSCs that is maintained after entry into meiosis. As is the case for a number of invertebrates, particularly during larval stages, the rapid growth of *Oikopleura* is underpinned by extensive deployment of endoreplicative cell cycle variants [17], [18]. Thus, this chordate model affords comparative opportunities to evaluate the extent of evolutionary conservation or innovation in developmental growth arrest mechanisms with respect to the signaling pathways employed, the links and controls in the longevity-reproduction axis, and the differential response of cell cycle variants in entry into, and release from, developmentally growth arrested states.

A number of conserved signaling pathways are important in coordinating the cellular and organismal response to environmental stresses such as starvation, and the resultant growth arrested states. These include the AMP-activated protein kinase (AMPK), insulin-like signaling, target of rapamycin (TOR), and the mitogen-activated protein kinases (MAPKs), of which the extracellular signal related kinases (ERKs) and p38 kinases are among the best characterized in response to stress. AMPK up-regulates catabolic pathways that generate ATP while down-regulating anabolic processes that consume ATP [19]. When AMPK activity is compromised in *C. elegans*, animals have reduced survival and fail to maintain mitotic quiescence in GSCs during L1 arrest [20]. They also develop into sterile adults when nutrition is restored. An important early finding in the genetics of ageing was that the lifespan of *C. elegans* was doubled by mutations in the phosphatidylinositol 3-kinase (PI3K) [21] or insulin receptor (InR) proteins [22], components of the insulin-like signaling pathway. This pathway has subsequently been demonstrated to modulate ageing in flies and mice [1]. Insulin-like signaling regulates the location and activity of the FOXO transcription factor, which translocates to the nucleus during growth arrest [23], [24].

The TOR pathway is central in mediating growth and reproduction in response to amino acid and growth factor availability, with multiple links to the signaling pathways mentioned above, which modulate coordinated metabolic response to dietary restriction. Inhibition of TOR extends lifespan in a number of species [25]. This operates through effects on i) transcription, the FOXO transcription factor is required for lifespan extension in response to TOR inhibition [26], ii) systemic effects on hormones, TOR regulates the expression of the insulin-like peptide *ins-7* [27] and iii) through non-transcriptional mechanisms including TOR-mediated phosphorylation of the S6 kinase (S6K) in the regulation of mRNA translation [25].

Loss of MAPK signaling also reduces life span and stress survival in *C. elegans* [28]. In calorie-restricted, long-lived mice, ERK has been shown to be constitutively active [29]. Similarly, in *C. elegans* and *Drosophila,* defects in the p38 pathway resulted in decreased survival abilities under starvation [30]–[32]. The ERK and p38 MAPK pathways are known to activate TOR and its downstream effectors [33]–[35]. Interestingly, inhibition of TOR activates MAPK to promote cell cycle progression in unicellular yeast [36] and mammalian cancer cell lines overcome TOR inhibition by activating ERK1/2 signaling [37], [38]. TOR inhibition also activates p38 signaling in megakaryocytes [39] and in glioblastoma cells [40]. The Mitogen and Stress Induced Kinase (MSK) has been identified as a common downstream effector of both ERK and p38 survival pathways [41]. MSK1 plays a major role in stress-induced epigenetic changes and regulation of transcription under stress [42].

In this study we show that growth arrest occurs in *Oikopleura dioica* in response to crowded conditions with nutrient limitation. Under such conditions, somatic endoreduplicative cell cycles were arrested prior to somatic mitotic or germline endomitotic proliferative cycles. Mitotic cycles also resumed prior to endocycles upon release from growth arrest. We further characterize the implication of TOR, and the MAPK, ERK and p38 signaling pathways, as well as central cell cycle transition regulators, Cyclin D and Cyclin-dependent Kinase Inhibitors (CKI) and the transcription factor E2F1, in growth arrest. Importantly, when exposed to crowded, nutrient-limited conditions, growth arrest in *O. dioica* only occurred in a post-metamorphic window prior to meiotic entry. Animals exposed to crowded, nutrient-limited conditions after meiotic commitment failed to arrest, instead maturing and spawning within a normal lifespan but with significantly reduced reproductive output.

## Materials and Methods

### *Oikopleura dioica* Culture and Growth Arrest

Collection and standard culture of *O. dioica* were conducted as previously [43]. Growth arrest (GA) was induced by initiating a culture of 3000 day 1 animals in a 6 liter beaker at 15°C and this was maintained for up to 18 days. Animals under GA conditions were fed twice daily with the standard algal strain mixture normally given at standard culture densities. The beakers were brushed every 12 h to remove discarded houses and other debris. Growth arrest was released by manual transfer of 150 GA animals from dense cultures into 6 liters of fresh sea water which was then maintained under standard culture conditions until maturity.

### Determination of Survival, Maturity, Fecundity and Hatching Success

To calculate survival, three independent volumes (200 ml) were sampled from each of three independent populations (biological replicates). Survival percentage was calculated as the (number of live animals)/(number of expected animals based on initial density)*100. At day 6, maturity was assessed based on the visual detection of mature gametes (Fig. S1) in a sample of 150–200 animals per population that was assessed. Fecundity and hatching success were calculated as described [44]. Student t-tests were carried out to assess the significance of any differences observed in each of the parameters.

### Manipulation of Animal Density at Different Developmental Time Points

To determine at which developmental stages GA could be induced, animals were initially cultured under standard conditions (50 animals/liter) and then exposed to higher density conditions (500 animals/liter) at either day 3, day 4 or day 5, all the time being maintained on a standard 1X algal diet. In each case, at day 6, survival, maturation, fecundity and hatching success of offspring were determined as above.

Fecundity and egg viability were also assessed in animals introduced to dense conditions at day 4 or day 5 and in animals released from GA through dilution to standard densities. In each treatment, eggs per female were counted and *in vitro* fertilization (IVF) was performed as follows. Sperm from 4–5 males cultured under standard conditions was collected in a petri dish and diluted in 5 ml sterile filtered sea water. An aliquot of 200–250 μl of this diluted sperm solution was added to freshly spawned oocytes in sterile-filtered sea water in a watch glass and incubated for 2.5 min followed by a wash with sea water. Fertilization rates and hatching success were then evaluated as above. For statistical analysis of fecundity and hatching success, 3 different populations were studied and for each population, the progeny of 10 females was used.

### Growth Measurements

To determine body lengths under the various experimental treatments, animals were fixed in 4% Paraformaldehyde (PFA)/0.1 M MOPS (pH 7.0)/0.5 M NaCl/5 mM EGTA/0.2% TritonX-100 at room temperature (RT) for 1 h. Body length was then determined using a Nikon SM1500 stereo microscopic equipped with a Nikon digital sight DS-5M camera. Measurement of body length was aided by the use Nikon Eclipse Net imaging software.

### Treatment with Pharmacological Inhibitors

For exposure to TOR inhibitor CCI-779 (LC chemicals), MAPK p38 inhibitor SB 203580 (LC chemicals), or MAPK ERK1/2 inhibitor UO126 (LC chemicals), cultures with animals at dense or standard conditions were supplemented with the inhibitors or equivalent volume of the DMSO solvent alone as a control, for 24 h at 15°C. Increasing concentrations of CCI-779 (5–20 μM), SB 203580 (5–30 μM), or U0126 (5–30 μM) were tested to define a working concentration which resulted in less than 20 percent mortality over a 24-h period. Selected final concentrations were 7.5 μM CCI-779, 15 μM SB 203580 and 5 μM UO126 and these experiments were carried out in 3-liter beakers. In response to these pharmacological treatments, either individually or in combination, survival and maturation were evaluated as above.

### RNA Isolation and Quantitative RT-PCR

Total RNA was isolated using RNeasy (Qiagen) from 250–300 animals, cultured at either dense or standard conditions, at day 3, day 4 and day 5. Following DNase treatment (Amp grade-Invitrogen), 1 μg of purified total RNA was used for making cDNA using MMLV reverse transcriptase enzyme (Invitrogen). Aliquots of 10 μl of 50-fold diluted cDNA were used to set up 20 μl PCR reaction mixes containing 500 nM each of forward and reverse primers (**Table. S1**) and 2X power SYBR green PCR master mix (Qiagen). Reactions were run in triplicates using the CFX 96 Real time system (Biorad). After initial denaturation for 5 min at 95°C, 40 cycles of 95°C for 15 s, 58°C for 20 s and 72°C for 20 s were conducted with final extension at 72°C for 5 min. Reverse transcriptase negative controls (reaction without MMLV enzyme) were run 40 cycles. Primer pair specificities were confirmed by melting curve evaluation, cloning, and sequencing of amplicons.

### Antibodies

Affinity purified polyclonal anti-Cyclin Dd (GDVDLKKFADHLSIPFEFLRC) [18], anti-CKIa (CPRSNLDTKVQKSAIKKS), and anti-E2F1 (SPSLFPSNVANQSVKMSK) antibodies were produced by 21^st^ Century Biochemical (Malbora MA). Primary antibodies anti-phospho 4EBP1, anti-phospho RPS6, anti-phospho MSK1, anti-phospho p38 (all from Cell Signaling), anti-phospho ERK1/2 (Millipore), anti-Histone H3 and, anti-phospho H3pS28 (Abcam), and anti-IdU and BrdU (Accurate Chemicals) were used for immunofluorescence staining and western blot analysis as described below.

### Immunofluorescence Staining

Animals were fixed in 4% Paraformaldehyde (PFA)/0.1 M MOPS (pH 7.0)/0.5 M NaCl/5 mM EGTA/0.2% TritonX-100 at 4°C overnight. Samples were then washed once with PBSTE (1X PBS, 1 mM EDTA, 0.1% TritonX-100) and three times with PBSTG (PBSTE+100 mM glycine) at room temperature (RT). Samples were blocked with 3% BSA in PBSTE at 4°C overnight. Incubation of primary antibodies (1∶100) was done in 3% BSA in PBSTE at 4°C for a minimum of 5 days. Samples were then washed 6 times with PBSTE at RT and post-fixed overnight in 4% PFA in PBSTE. Samples were then washed once with PBSTE, three times with PBSTG and twice with PBSTE. Following 4–6 days of secondary antibody incubation at 4°C, samples were washed 6 times with PBSTE at RT. DNA was counterstained with 1 μM To-Pro-3 iodide (Molecular Probes) and mounted in vector shield (Vector Laboratories). Samples were analyzed by confocal microscopy using a Leica TCS laser scanning confocal microscope and Leica (LAS AF v2.3) software. Anti-rabbit and anti-rat Alexa488 (Molecular Probes), and anti –mouse 488 and anti-mouse 568 (Invitrogen) secondary antibodies were used at 1∶500 dilution.

### Replication Pulse Labeling

Replication single and double pulse labeling using IdU and CldU (Sigma), were performed as previously [18], [45]. For single IdU pulse experiments, animals were transferred to fresh sea water containing 0.5 mM IdU in a watch glass and incubated with mild shaking for 45 min. Subsequent fixation was done in 4% PFA, at 4°C overnight. In double pulse experiments, GA animals or GA animals released into standard density for 2 or 3 h, were pulsed with CldU for 1 h followed by washing with fresh sea water and subsequent pulsing with IdU for 1 h, all at 15°C. Fixed animals were washed once with PBST (PBS, 0.2% Tween 20) and three times with PBSTG, followed by incubation with DNaseI (5 U/ml amp grade, Invitrogen) in PBSTG, containing 0.5% bovine serum albumin (BSA), and 1 mM MgCl_2_ for 1 h at RT. The reaction was stopped by adding PBSTG containing 10 mM EDTA, followed by six washes in PBSTE. The samples were blocked overnight with 3% BSA at 4°C and processed for immunostaining as above. In double pulse experiments, CldU and IdU were detected by sequential incubations with rat monoclonal anti-BrdU (clone Bu1/75-Accurate Chemicals), which reacts with CldU but not with IdU, and a mouse monoclonal anti-IdU which binds preferentially to IdU (clone IU-4-Accurate Chemicals). After primary antibody incubation, post fixation was done with 4% PFA in PBSTE overnight at 4°C. Further sample processing was as above.

### Whole Cell Lysate Preparation and Western Blotting

Animals collected in fresh seawater were washed once with ice cold PBS prior to snap freezing in liquid nitrogen for future analysis. Whole cell lysates were prepared either from fresh or frozen animals by boiling in 1X Laemmli sample buffer (50 mM Tris pH6.8, 2% sodium dodecyl sulfate (SDS), 10% glycerol, 250 mM β–mercaptoethanol, 0.001% bromophenol blue) at 95°C for 10 min followed by vortexing and centrifuging 5 min at 13000 g to remove cellular debris from the lysate. Equal amounts of protein were applied to 10–12% SDS Polyacrylamide gel and transferred to nitrocellulose membranes (Whatman) in 1X transfer buffer (250 mM Tris, 190 mM glycine, 0.5% SDS, 20% methanol). Post-transfer, the membrane was rinsed with 1X Tris Buffer Saline (TBS- 50 mM Tris-Cl, pH 7.6; 150 mM NaCl) and blocked for a minimum of 1 h in 3% BSA in 1X TBST (1X TBS +0.1% Tween 20) at RT. The membrane was incubated with primary antibodies (1∶1000 dilution, except for anti-phospho ERK1/2 at 1∶2000, and anti-Histone H3 at 1∶10000) overnight at 4°C in 3% BSA/TBST. Following primary antibody incubation the membrane was washed three times with TBST at RT followed by incubation with HRP-conjugated secondary antibodies (Goat anti-rabbit from Zymed, 1∶10000) in 3% BSA/TBST for 1 h at RT. Following washes, HRP generated chemiluminescent signal was detected using either the Chemi Doc XRS+ image system (Biorad) or X-ray film after addition of the ECL rapid chemiluminescent detection reagent (Calbiochem).

## Results

### High Density Culture Induces Reversible Growth Arrest (GA) and Prolongs the Life Span of *Oikopleura dioica*

**Figure 1:**
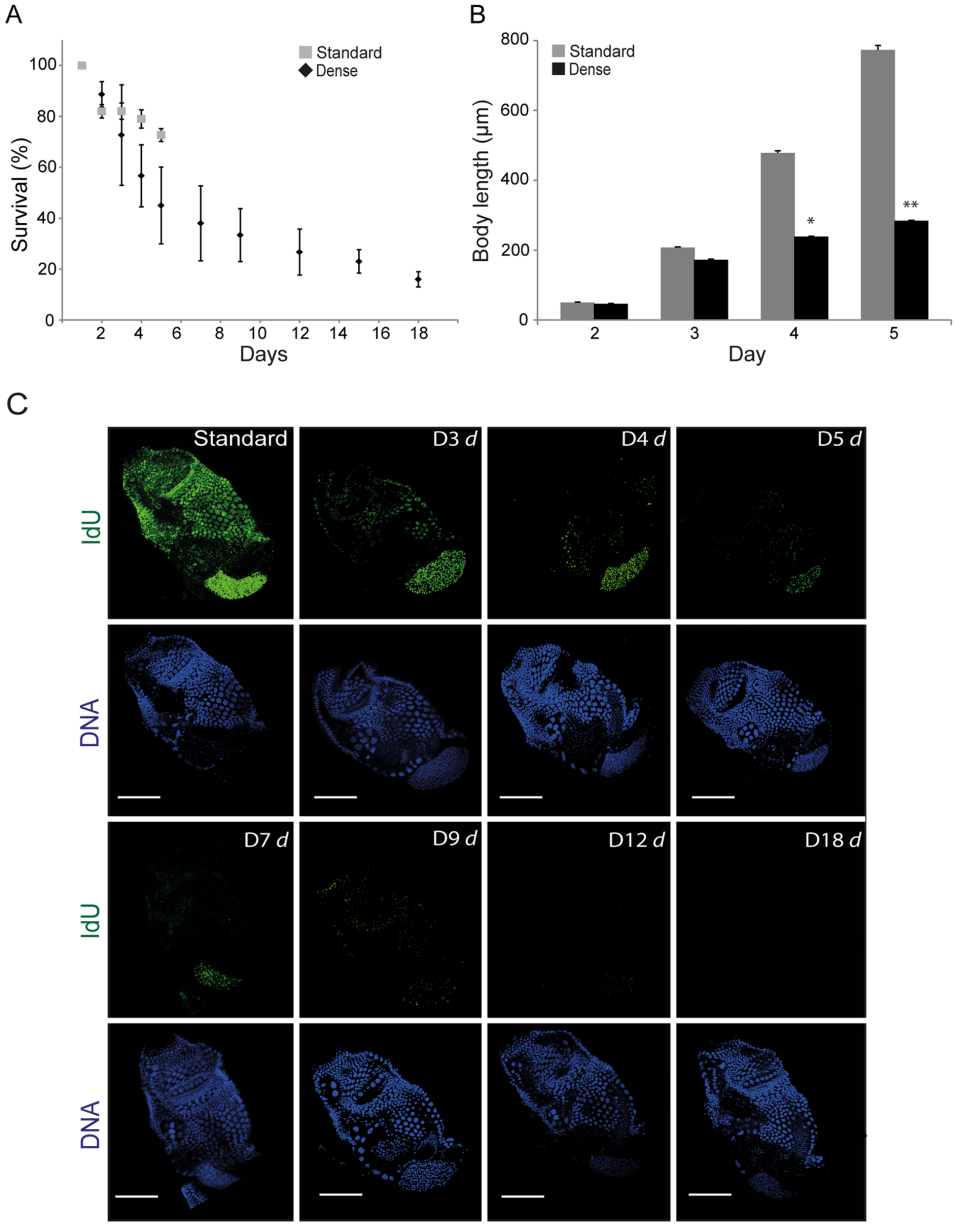
Growth arrest in *Oikopleura dioica*. A) At standard densities *O. dioica* completes its life cycle in 6 days at which point it spawns and dies. At higher culture densities *O. dioica* undergoes growth arrest (GA) and the animal can exhibit a 3-fold increase in lifespan, albeit, with increasing mortality up to day 18. B) At high culture densities GA animals undergo very limited increase in body length compared to control animals at standard culture densities (significant differences: *p<0.05, **p<0.01). The morphology of surviving GA animals throughout the 18–day period remained similar to that of day 3 animals cultured under standard conditions. Error bars indicate standard errors in A and B. C) A rapid post-day 3 reduction in IdU incorporation (S phase marker) was observed in somatic endocycling cells in animals cultured under dense conditions. IdU incorporation persisted longer in mitotically proliferating germ nuclei in these same animals but eventually diminished as growth arrest persisted (D3 *d* = day 3 dense etc.). Scale bars = 50 μm.

Under standard laboratory culture densities (25 individuals L^−1^) *Oikopleura dioica* completes its life cycle within 6 days at 15°C [43]. At higher culture densities (500 individuals L^−1^), it undergoes GA and maintains this state for more than 18 days (**Fig. 1A**). During this period, survival diminished, but animals that did survive retained the morphology and size of day 3 pre-meiotic animals. *O. dioica* cultured at high densities grew similarly to those at standard densities up to day 3 but subsequently exhibited very little increase in size (**Fig. 1B**). The normally rapid growth of *O. dioica* occurs by increasing the size of somatic cells through endocycles (**Fig. S2**). As the animal reaches maturity (early day 6), somatic endocycling ceases, whereas cell cycle progression continues in the gonads, with a concomitant increase in gonad size relative to the trunk [17]. During day 6 at standard density culture conditions, the animals spawn and die. When *O. dioica* were cultured at high density, there was a rapid reduction of IdU incorporation in somatic endocycling cells from day 3 onwards (**Fig. 1C**) with full arrest attained at day 5 of culture under these dense conditions. IdU incorporation persisted longer, and at higher rates, in endomitotic germline nuclei but also gradually decreased to barely detectable levels by day 12 of dense culture.

### Upon Release from Growth Arrest, Germline and Somatic Mitotic Cycles are Re-activated before Somatic Endocycles

**Figure 2:**
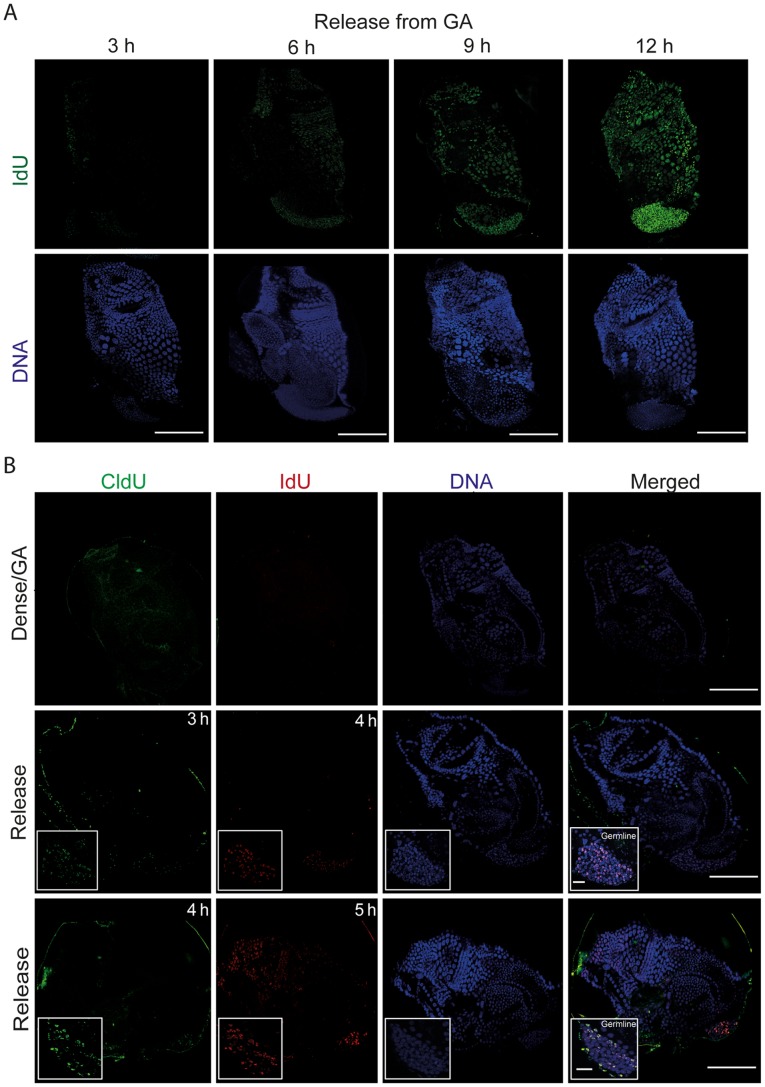
Following release from growth arrest, mitotic germline nuclei re-enter S-phase before somatic endocycling cells. A) IdU (S-phase marker) incorporation was gradually restored in the endocycling epithelial and mitotic germ line nuclei of GA animals that had been released into standard culture conditions. Scale bars = 50 μm. B) Pulse chases of CldU and IdU (see also materials and methods and Fig. S3) in GA animals (top panels), or in animals that had been released from GA for 2 (mid-panels) or 3 h (bottom panels), revealed that resumption of S-phase in the mitotic germline preceded that in somatic endocycling cells by 1 h. Scale bars = 50 μm. Insets depict zooms of the germline (Scale bars = 10 μm).

Given the differential response of somatic endocycles and germline endomitotic cycles to dense culture conditions, we next asked how these different cycles would respond to release from GA. To do this, we transferred GA animals into standard culture densities and over an interval of 2 to 11 h post-release, exposed them to a 1 h pulse of IdU. This revealed that re-entry into S–phase began at least as early as 6 h post-release and was fully re-established in both cell cycle types within 12 h post-release(**Fig. 2A**). To determine which cell cycle type first re-entered S-phase upon release from GA, animals were released into standard culture conditions for 2 or 3 h and pulsed first with CldU for 1 h followed by a pulse of IdU for 1 h (see also **Fig. S3**). Within the first 4 h of release into standard densities, the S-phase marker was first restored in the endomitotic germline (**Fig. 2B**) and then mitotic intestinal cells (**Fig. S3**). Within 5 h of release, arrested somatic endocycling cells had re-entered S-phase. This showed that the germline endomitotic cell cycle, which undergoes a delayed arrest upon entry into GA, is also the most rapidly re-activated in response to improved nutritional cues, followed by somatic mitotic cycles and finally, somatic endocycles.

### Inhibition of TOR Signaling, or Nutrient Limitation, Mimic Growth Arrest in *Oikopleura dioica* Cultured at High Density

**Figure 3:**
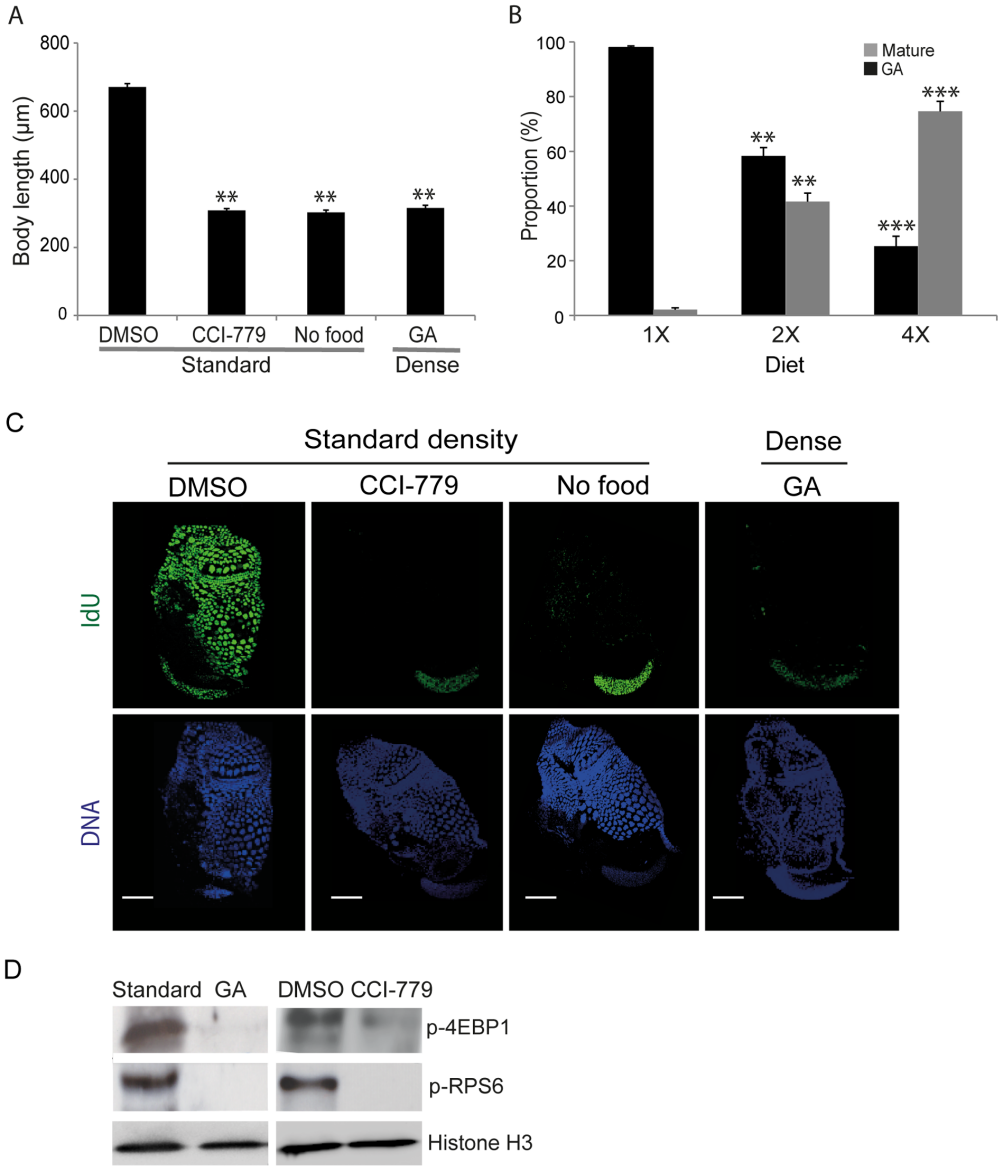
Inhibition of TOR signaling or dietary restriction mimics growth arrest in *Oikopleura dioica.* A) When *O. dioica* was cultured at standard densities in the 24 h presence of the TOR inhibitor CCI-779 (7.5 μM) or 24 h in the absence of food, a significant (**p<0.01) growth arrest (GA) similar to that identified under dense conditions was observed. B) When animals were cultured under dense conditions, GA was alleviated by increasing food availability in a dose-dependent manner. Significant differences (**p<0.01, ***p<0.001) in GA and maturation at 2X and 4X diets are indicated with respect to those animals maintained at a 1X diet under dense conditions. Error bars in A and B indicate standard errors. C) *O. dioica* cultured under standard conditions in the presence of 7.5 μM TOR inhibitor CCI-779 (rapamycin analog) for 24 h or animals cultured without food for 24 h also showed arrest of somatic endocycles whereas mitotically proliferating germ nuclei continued to cycle. Scale bars = 50 μm in A and B. D) Western blots showed that TOR signaling was inhibited in GA animals cultured under dense conditions as it was in animals cultured at standard densities in the presence of the TOR inhibitor CCI-779. Activation of the downstream effectors of TOR signaling, 4EBP1 and RPS6, was reduced under both of these treatments as compared to animals cultured at standard densities, in the presence or absence of DMSO.

To compare GA in *O. dioica* with the effects of inhibiting TOR signaling or restricted nutritional input, body length was measured in animals cultured at standard densities in the presence of TOR inhibitor CCI-779 or in the absence of food for a 24 h period. In both cases growth was arrested to a level similar to that observed under dense culture conditions (**Fig. 3A**). We then asked whether GA under dense conditions was principally a result of reduced nutrition. Growth and maturity in *O. dioica* were assessed under dense conditions but with diet regimes ranging from 1-fold to 4-fold of the standard diet. Increased food under dense conditions reduced GA and increased sexual maturation in a dose-dependent manner (**Fig. 3B**). To attain standard diet levels per individual at dense conditions would require culturing the animals with 20 times the standard food concentrations. It has been shown previously that animals cultured at standard densities, but with acutely limited nutrition, do not enter GA, but instead produce very limited or no gametes [46]. It is also known that filter-feeding *O. dioica* cultured in the presence of high concentrations of food particles experience much higher mortality rates due to filter clogging [43], requiring rates of house replacement exceeding the physiological capacity of the animal. We observed such increased mortality under dense conditions when the food concentration was increased to 8-fold the normal diet. Although we cannot conclude that nutrient limitation was the sole factor driving GA, the observation that a 4-fold increase in food concentration (versus a 20-fold increase in animal density) alleviated GA by 75% (Fig. 3B), indicates that it is a significant factor. Thus in *O. dioica*, GA occurs under high population density where nutrition is limited.

With respect to the differential response of somatic endocycles and germline endomitotic cycles to GA (**Fig. 1C**), similar results were obtained when day 2 to day 3 *O. dioica* were cultured at standard density, but: i) for 24 h in the presence of a Rapamycin analog CCI-779, known to block the TOR signaling pathway, or ii) in the absence of food for 24 h (**Fig. 3C**). These findings indicate that GA in *O. dioica* involves inhibition of TOR signaling in response to diminishing nutrient availability in dense cultures.

To test if there was a reduction in TOR activity in GA animals, the phosphorylation of TOR signaling downstream effectors 4EBP1 and RPS6 was compared by western blotting of whole animal lysates from those in GA, or those cultured in standard conditions in the presence or absence of DMSO or CCI-779 for 24 h (**Fig. 3D**). Phosphorylation of 4EBP1 and RPS6 was reduced in both GA animals or those treated with CCI-779, confirming the involvement of TOR signaling in regulating GA in *O. dioica.*

### Growth Arrest, or TOR Inhibition, Block Meiotic Onset in the Germline

**Figure 4:**
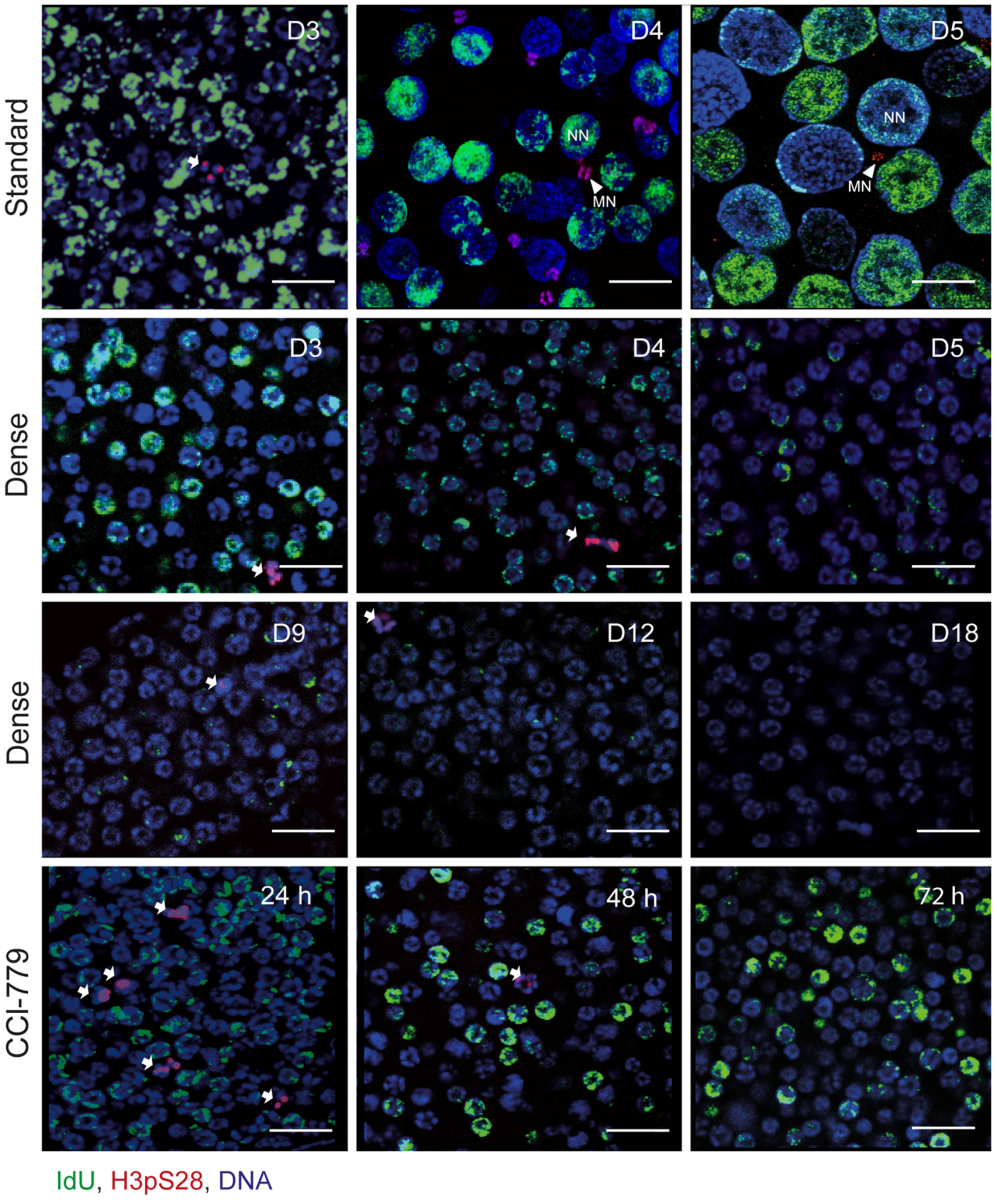
Inhibition of TOR signaling prevents meiotic onset in *Oikopleura dioica*. At standard culture densities (top row), the *O. dioica* germline is characterized by a coenocystic cytoplasm consisting of a homogenous population of proliferative mitotic nuclei prior to day 3 of development. Following meiotic onset, (late day 3) the germline coenocystic cytoplasm consists of equal numbers of smaller meiotic nuclei (MN, arrowheads) arrested in prophase I and larger endocycling nurse nuclei (NN). Germline nuclei of growth arrested animals (Dense condition) retain a homogeneous undifferentiated size and do not commence meiosis as evident from the persistence of the proliferative mitotic mark H3pS28 (arrows) similar to Day3 animals before meiotic onset under standard conditions. There was a decrease in IdU incorporation in germline nuclei over time (D3–D18; day 3 to day 18). Similarly, germ line nuclei of *O. dioica* cultured at standard density, do not commence meiosis when treated with the TOR inhibitor CCI-779 (7.5 μM) for 24 h, 48 h or 72 h and continue to proliferate mitotically. Scale bars = 20 μm.

During the first half (up to day 3) of the *O. dioica* life cycle, endomitotic proliferation is evident in undifferentiated syncitial germline nuclei and mitotic proliferation is observed in intestinal cells [15], [17]. At later stages, with the onset of meiosis, the female germline is characterized by equal numbers of small meiotic nuclei arrested in prophase I and asynchronously endocycling nurse nuclei, all of which share a common cytoplasm in one giant cell referred to as the coenocyst [14]–[16]. To examine possible roles of TOR signaling in germline proliferation and meiotic onset, GA animals (Dense) were assessed for S-phase IdU incorporation and immunostained for the mitotic marker H3pS28 [47] (**Fig. 4**). Pre-meiotic germline nuclei continued to incorporate IdU during the early days of GA but gradually ceased incorporation as GA persisted to day 18. Interestingly, germline nuclei in GA animals do not enter meiosis as is evident by their retention of a homogenous size, equivalent to that found in day 3 animals cultured under standard conditions, and the persistence of mitotic marker histone H3pS28.

Persistence of endomitotic proliferation of syncitial germline nuclei without entry into meiosis was also observed in animals cultured at standard density when the TOR inhibitor CCI-779 was introduced at day 3. Under these conditions, endomitotic proliferation continued until day 6, beyond which animals did not survive (**Fig. 4**, bottom panels). Additionally, the proliferation of other mitotic cell types in *O. dioica* such as the intestinal cells was not affected immediately in GA animals, or in animals cultured at standard density in the presence of CCI-779 or in the absence of food (**Fig. S3**). These results show that TOR signaling in *O. dioica* differentially impacts cell cycle progression in somatic endocycles versus mitotic cycles, as seen in *Drosophila* and *C. elegans* [7], [48]–[50].

### MAPK ERK1/2 and MAPK p38 Pathways are Activated and Important for Survival of Growth-arrested *Oikopleura dioica*

**Figure 5:**
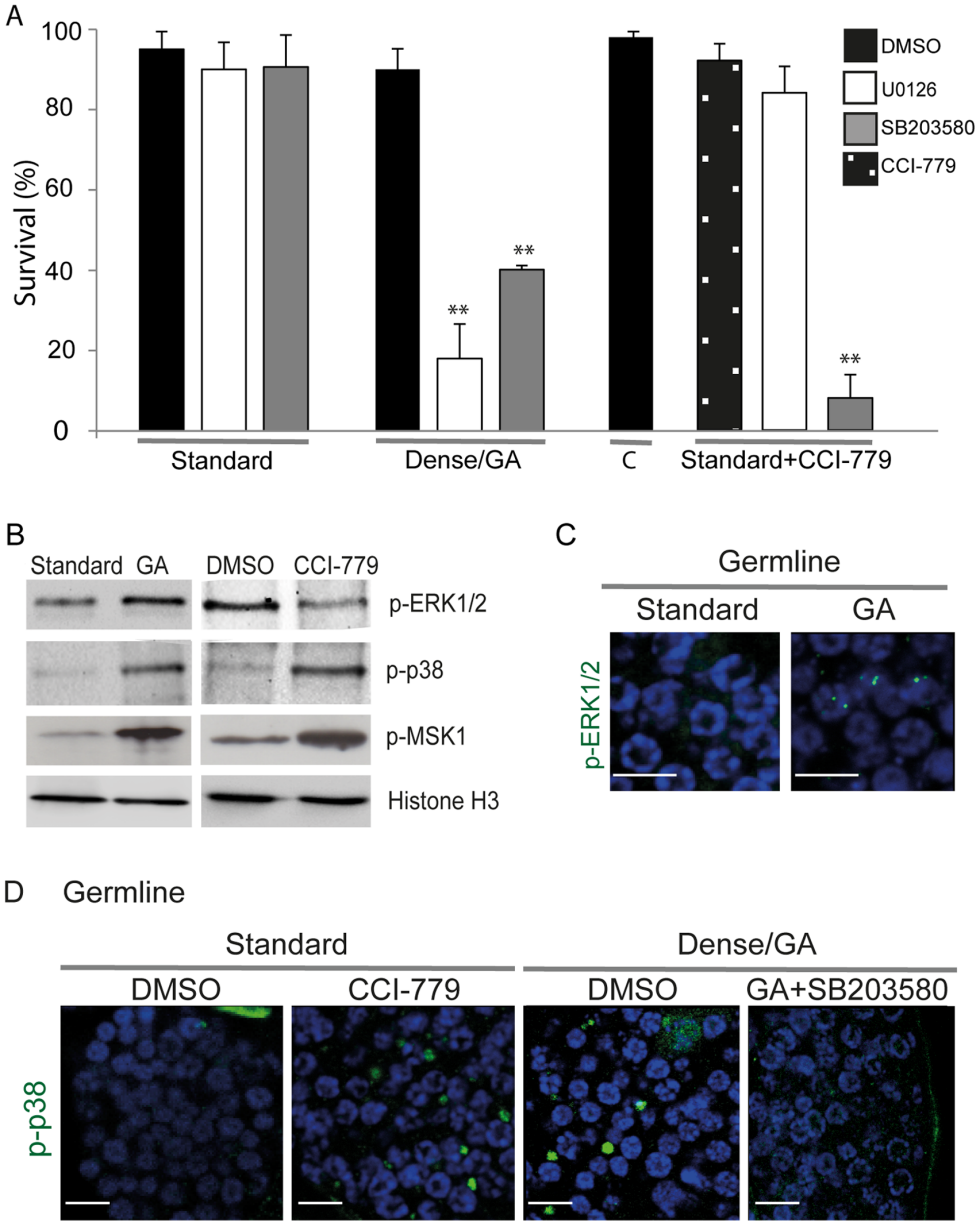
Activation of MAPK kinases ERK1/2 and p38 in the survival of growth-arrested *Oikopleura dioica*. A) When *O. dioica* was cultured under standard conditions in the presence of the ERK1/2 inhibitor U0126 (5 μM) or the p38 inhibitor SB203580 (20 μM) there were no effects on survival. In contrast, the survival of GA animals was markedly reduced (**p<0.01) in the presence of the same individual inhibitors at the same concentrations. Under standard culture conditions, the combined pharmacological inhibition of TOR by CCI-779 (7.5 μM) and ERK1/2 by U0126, did not affect survival over a 24 h period, whereas CCI-779 combined with SB203580 inhibition of p38 over the same period, substantially reduced survival (**p<0.01). “C” = control animals for this single versus combined inhibitor experiment cultured only in the presence of DMSO. Error bars indicate standard error. B) Western blots of whole cell lysates from animals cultured at high or standard densities, or at standard densities, with and without CCI-779 treatment. Both ERK1/2 and p38 survival pathways and their common downstream effector MSK1 were activated in GA animals. In contrast, pharmacological inhibition of TOR activated only p38 and MSK1 but not ERK1/2. C) Immunostaining of phospho-ERK1/2 in the germline of GA animals revealed foci of phospho-ERK1/2 in nuclei. D) Immnostaining of phospho-p38 in the germline of GA and CCI-779 treated animals showed the presence of phospho-p38 as distinct cytoplasmic foci within the coenocystic germline. These phospho-p38 foci disappeared when GA animals were treated with the p38 inhibitor SB203580. Scale bars in C and D = 10 μm.

To determine whether MAPK pathways ERK1/2 and p38 were activated during GA, *O. dioica* were exposed to ERK1/2 inhibitor UO126 or p38 inhibitor SB 203580 under standard and dense culture conditions. Inhibition of MAPK, ERK1/2 or p38, under standard culture densities did not affect the survival of *O. dioica*. However, inhibition of ERK1/2 or p38 under dense, nutrient-limited, culture conditions (when TOR signaling is inhibited) reduced survival significantly (**Fig. 5A**), indicating that the MAPK survival signaling pathways, ERK1/2 and MAPK p38, were activated under these conditions in *O. dioica*. Animals were unable to survive when both the TOR signaling and MAPK p38 pathways were blocked through exposure to both CCI-779 and SB203580, respectively. In striking contrast, survival of *O. dioica* was not affected by combined inhibition of TOR signaling and MAPK ERK1/2 pathways.

To further characterize the involvement of MAPK ERK1/2 and p38 survival pathways, lysates of GA or CCI-779-treated *O. dioica* were western blotted to detect active forms of the kinases: phospho ERK1/2, phospho p38 and phospho MSK1. Interestingly, under GA, ERK1/2, p38 and MSK1 were all activated (**Fig. 5B**). However, when TOR signaling was inhibited by CCI-779 under standard culture densities, only p38 and MSK1 but not ERK1/2 exhibited increased phosphorylation. Whereas phospho-ERK1/2 appeared as foci in nuclei of the endomitotic germline under dense, nutrient-limited conditions (**Fig. 5C**), phospho-p38 appeared as foci in the germline cytoplasm under both dense conditions or during exposure to CCI-779 (**Fig. 5D**). When GA animals were exposed to the p38 inhibitor SB203580, these cytoplasmic foci disappeared (**Fig. 5D**). Thus, upon inhibition of TOR signaling alone, activation of p38 but not ERK1/2, was critical to survival.

### TOR Signaling Regulates Somatic Endocycling via *Oikopleura dioica* Cyclin Dd and Cyclin-dependent Kinase Inhibitor, CKIa

**Figure 6:**
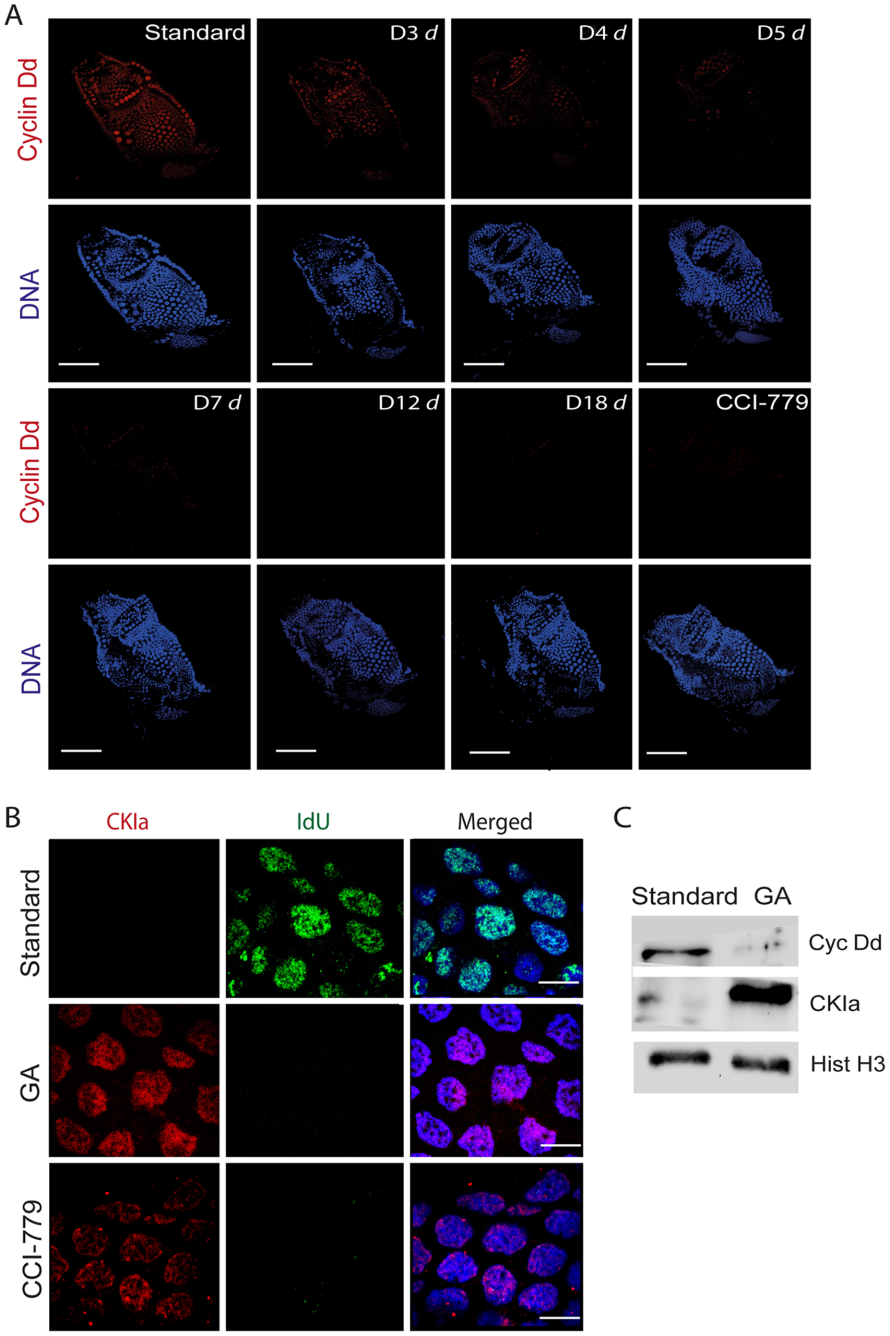
Growth arrest and TOR signaling act through G1 regulators, Cyclin Dd and CKIa, in somatic endocycles. A) A reduction in protein levels of Cyclin Dd, a G1 regulator, was observed in endocycling somatic cells under dense GA conditions (D3 *d = *day3 dense etc.). *O. dioica* cultured under standard conditions in the presence of CCI-779 (7.5 μM) for 24 h also showed reduction of Cyclin Dd expression in somatic endocycling cells. Scale bars = 50 μm. B) Cyclin-dependent Kinase Inhibitor a (CKIa), increased in concentration both in the arrested endocycling nuclei of GA animals as well as in the arrested somatic endocycling nuclei of the CCI-779 treated animals cultured at standard densities. DNA replication was simultaneously curtailed under both conditions as indicated by levels of IdU incorporation. Scale bars = 10 μm. C) Western blots of whole cell lysates from animals cultured at standard densities or in GA showed that in the latter case, Cyclin Dd levels were reduced, whereas CKIa levels were increased.

Inhibition of TOR signaling led to the rapid arrest of somatic endocycles (**Fig. 3C**). We therefore examined the effects of manipulating TOR signaling on the G-S transition cell cycle regulators Cyclin Dd and Cyclin-dependent Kinase Inhibitor a (CKIa) (see also **Fig. S4**). TOR signaling has been shown to positively regulate Cyclin D protein levels by regulating mRNA translation [51]. The relative expression of transcripts for the major D-type cyclin, Cyclin Dd, in somatic endocycles [18] did not change significantly in densely cultured nutrient-limited animals compared to those maintained in standard conditions either prior to or post day 3 (**Fig**. **S5A**). However, immunofluorescent staining of Cyclin Dd in GA animals, showed a reduction of Cyclin Dd (**Fig**. **6A**) levels in somatic endocycling cells that coincided with the observed reduction in IdU incorporation (S-phase entry) (**Fig**. **2A**). Similarly, animals at standard density cultured in the presence of TOR inhibitor CCI-779 also rapidly down-regulated Cyclin Dd levels in somatic endocycling cells (**Fig. 6A**). These results indicate that when TOR signaling is inhibited, either the translation of Cyclin Dd mRNA is reduced or the cyclin D protein is destabilized in somatic endocycling cells and that the inhibition of TOR that occurs during GA, down-regulates Cyclin Dd levels, with concomitant arrest of the somatic endocycle. Interestingly, ERK1/2 and p38 have also been shown to modulate the stability of cyclin D in cell culture [52].

In direct contrast to the above results, transcript levels of an antagonist of CyclinD-CDK activity, CKIa, were up-regulated at day 4 in GA animals cultured under dense nutrient-limited conditions compared to morphologically equivalent day 3 animals in standard culture conditions (**Fig. S5B**). CKIa levels increased strongly in the nuclei of somatic endocycling cells arrested in G-phase in response to GA or CCI-779 inhibition of TOR signaling (**Fig. 6B**). Thus, *CKIa* transcription and localization were governed by TOR signaling in somatic endocycling cells. In mammalian cells, TOR signaling is known to destabilize the CKI, p27, thus stimulating entry into S-phase [38], [53]. In *O. dioica*, inhibition of TOR signaling during GA simultaneously upregulated CKIa transcripts and protein levels while reducing Cyclin Dd protein levels, as somatic endocycles arrested (**Fig. 6C**).

### E2F1 Levels Exhibit Cell Cycle Oscillations in Germline Endomitotic Cycles Whereas they do not in Somatic Endocycles

**Figure 7:**
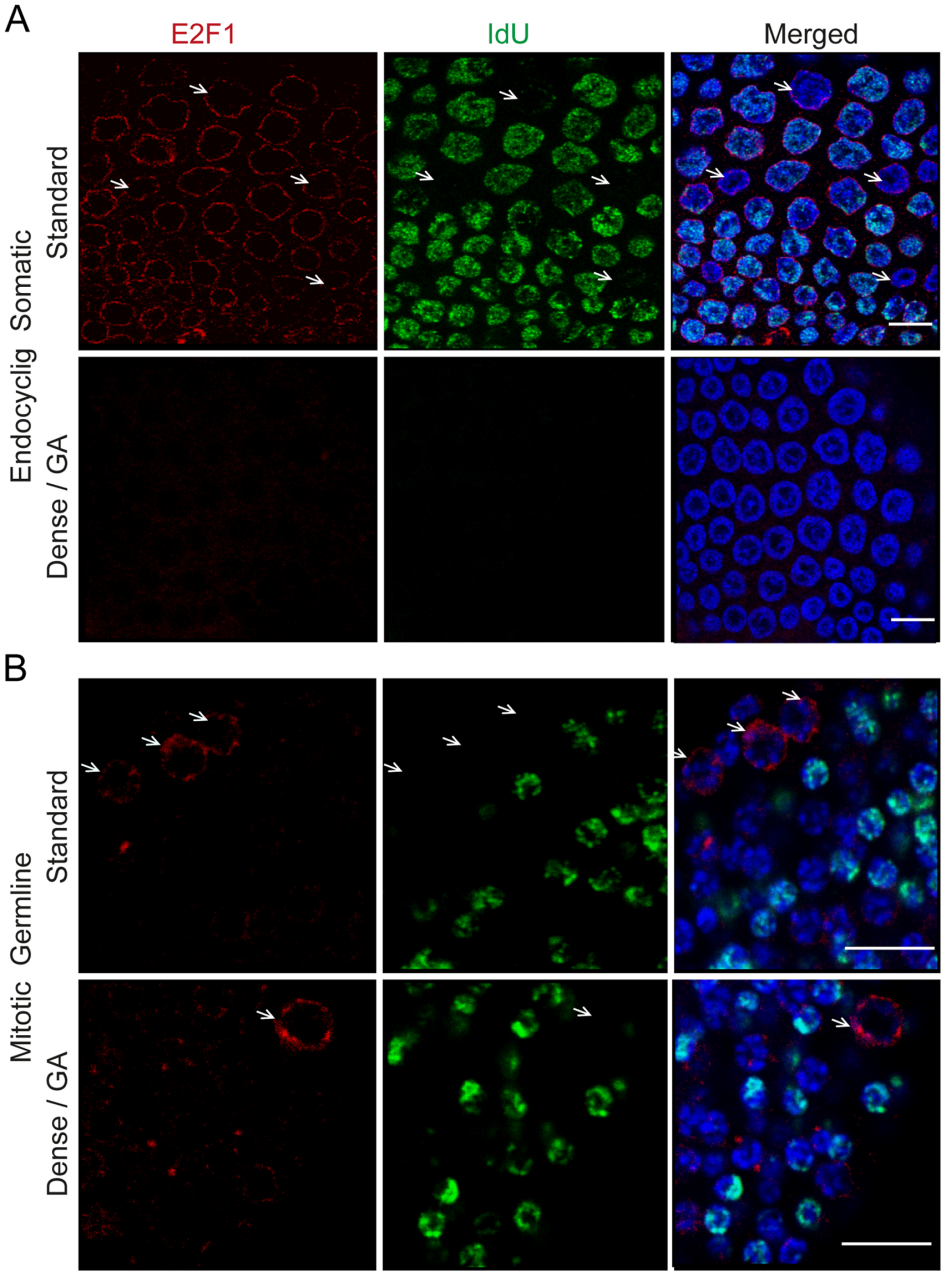
Levels of *O. dioica* E2F1 decline differentially in somatic endocycling cells versus mitotic germline cells during growth arrest. A) In somatic endocycling cells, E2F1 was constitutively present during both G- (arrows) and S-phases, under standard culture conditions. E2F1 was rapidly reduced in somatic endocycling cells during GA. This reduction coincided with a lack of incorporation of the S-phase marker IdU. B) Under standard culture conditions, E2F1 levels peaked during G1 (arrows) in mitotic germ line nuclei and were greatly reduced during S-phase. E2F1 levels persisted longer in the mitotic germline of GA animals than in somatic endocycling cells. Scale bars = 10 μm.

The E2F family of transcription factors are key regulators in promoting or repressing cell cycle progression [54]. In *Drosophila,* TOR inhibition reduces protein levels of E2F1 without affecting transcript expression, resulting in the arrest of endocycles. [55]. We examined the transcript and protein expression profiles of *O. dioica* E2F1 (**Fig. S6**) under both standard culture and GA conditions. Quantitative PCR analysis of E2F1 mRNA did not reveal a significant change in transcript expression during GA (**Fig. S5C**), but levels of E2F1 protein were rapidly reduced in somatic endocycling cells (**Fig. 7A**). This suggests that TOR signaling regulates the expression of E2F1 at the level of mRNA translation and not at the level of mRNA transcription in GA animals, similar to observations in *Drosophila*. It has been shown that the cyclical expression and degradation of E2F1 in the salivary glands of *Drosophila* drives endocycle progression, wherein E2F1 rises in G phase and is degraded in S phase [55]. Interestingly, immunostaining of E2F1 along with S phase marker IdU incorporation showed that in *O. dioica*, E2F1 levels also rose in G phase and dropped in S phase of the endomitotic germ line (**Fig. 7B**). Surprisingly, however, E2F1 appeared to be constitutive during both G- and S-phase in somatic endocycling cells of *O.dioica* (**Fig. 7A**). This suggests that oscillation of levels of E2F1 is important in regulating cell cycle progression in endomitotic germline nuclei but less so in somatic endocycling cells in *O. dioica*. Also, whereas E2F1 levels were immediately reduced in somatic endocycles upon GA, this was not the case in the endomitotic germline (**Fig. 7B**).

### Growth Arrest only Occurs Prior to Meiotic Entry

**Figure 8:**
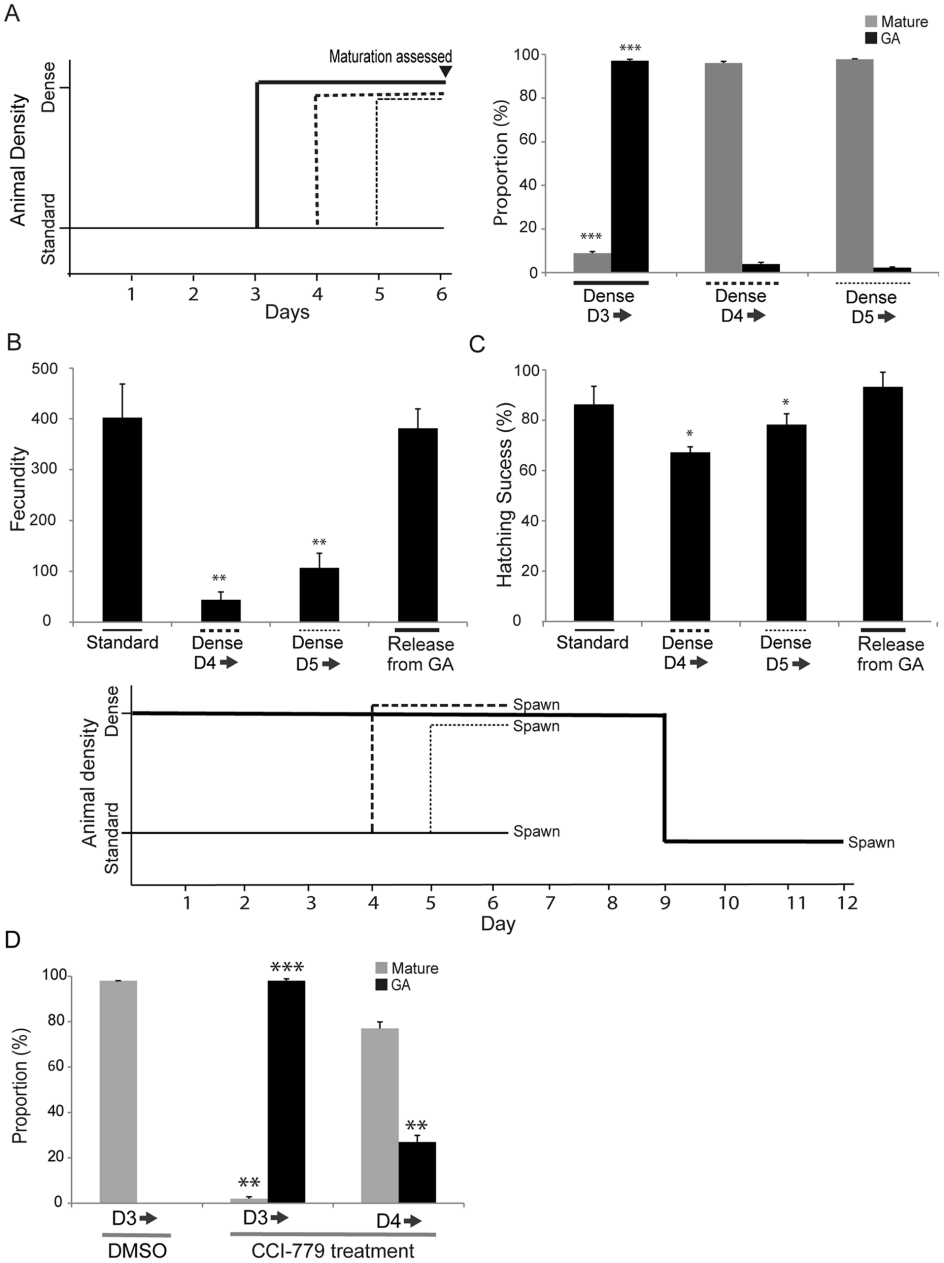
Growth arrest only occurs prior to meiotic entry. A) *O. dioica* only undergoes GA if exposed to dense, nutrient-limited conditions prior to day 4 of development under standard conditions. The schema at the left summarizes the experimental design. Animals were cultured under standard conditions and then introduced to dense culture conditions at either day 3, 4, or 5. GA was very significantly enhanced in those experiencing dense conditions prior to day 4 (Dense D3 ) compared to those exposed from day 4 or 5 (***p<0.001, with respect to normally developing animals at standard density). Maturation was significantly reduced in animals exposed to increased density prior to day 4 whereas those exposed later matured with the same timing as animals maintained throughout the experiment under standard culture conditions. B) Animals released from GA by dilution to standard density complete maturation and spawning in 2.5–3.5 days, a time frame similar to that required for animals with a day 3 morphology cultured under standard conditions. The fecundity of animals released from GA was not significantly affected compared to animals cultured under standard conditions throughout, whereas in contrast, animals cultured initially under standard conditions and then transferred to higher density at day 4 or 5, exhibited significantly reduced fecundity (**p<0.01). C) In addition to reduced fecundity, there was also a slight reduction (*p<0.05) in the quality of the eggs produced by animals exposed to dense conditions from day 4 or 5 as judged by their capacity to generate embryos that developed and hatched successfully with normal morphology. The schema shown below panels B and C depicts the experimental design corresponding to both of these result panels. D) Inhibition of TOR signaling in the presence of CCI-779 was more efficient in establishing GA and preventing animal maturation when introduced from day 3 onwards (D3 ), prior to initiation of meiosis in females, than it was when introduced from day 4 onwards, after meiotic entry. Significant differences in GA and maturation are indicated with respect to animals cultured at standard density in the presence of DMSO from day 3 onwards. Error bars indicate standard error.

We established above that animals cultured under dense conditions from the start of the life cycle did not enter GA until day 3. To determine if GA could also be induced at subsequent stages, animals were cultured at standard densities and transferred to dense conditions at various time points (**Fig. 8**). More specifically, since meiosis commences in *O. dioica* females at the day 3 to 4 transition [14], [15] we were interested as to whether GA could be induced both prior to and post commencement of meiosis. Animals were cultured at standard densities to day 3, 4 or 5 and subsequently placed in dense conditions (**Fig. 8A**). We only observed GA in animals exposed to dense conditions prior to day 3. Those exposed later exhibited no difference in maturation as compared to animals maintained at standard culture densities throughout.

When animals in dense cultures at GA were diluted to standard culture densities they rapidly resumed a normal progression and completed the life cycle in 2.5 to 3.5 days post–release, a time frame for maturation similar to that required from the mid-point of the life cycle for animals maintained in standard conditions throughout. The fecundity of animals released from a GA day 3-morphology was similar to that of animals completing a normal 6-day life cycle under standard culture conditions (**Fig. 8B**). On the other hand, animals introduced to dense conditions at day 4 or 5, matured and spawned as usual at day 6 but exhibited significantly reduced fecundity.

We then evaluated the quality of embryos produced under these different conditions as judged by hatching success. Animals cultured under dense conditions prior to day 3, which entered GA and were then released into standard culture conditions, and allowed to mature and spawn, produced progeny that exhibited similar rates of hatching success as did the progeny of animals maintained under standard culture densities throughout the life cycle (**Fig. 8C**). In comparison, there was a slight, but significant, reduction in the hatching success of progeny derived from animals introduced to dense conditions at day 4 or 5 of the life cycle. Thus, *O. dioica* released from GA are capable of undergoing maturation and spawning to give rise to progeny that develop normally. On the other hand, animals exposed to dense conditions after day 3 are unable to enter GA, and instead, produce fewer oocytes of slightly inferior quality. Similar results were observed with respect to the direct inhibition of TOR signaling. Day 3 (pre-meiotic) and day 4 (post-meiotic onset) animals were cultured at standard densities in the presence of CCI-779. Day 3 animals introduced to CCI-779 survived until day 6 but failed to mature or exhibit gamete differentiation during this period (**Fig. 8D**). O the other hand, when day 4 animals were exposed to CCI-779, 75% of the animals matured at day 6. Inhibition of TOR signaling at day 3 was also very efficient in establishing GA whereas it was much less efficient in doing so when exposure occurred at day 4. Thus, inhibition of TOR signaling efficiently initiates GA and blocks maturation if it occurs prior to the onset of meiosis in the female germ line.

## Discussion

### Iteroparous and Semelparous Growth Arrest Models

In addition to a semelparous as opposed to iteroparous mode of reproduction, *Oikopleura dioica* also differs substantially in two central aspects of female reproductive organization from the well-studied *Drosophila* and *C. elegans* models. In the *Drosophila* germarium, at the anterior end of the ovariole, somatic cap cells and escort stem cells send signals to maintain a juxtaposed population of GSCs [56]. Entry into meiosis and maturation of oocytes then occurs in progressively more posterior locations along the ovariole. Similarly, in hermaphroditic *C. elegans*, the gonad is formed by two U-shaped tubes connected to a common uterus [57]. At the distal end of each tube, a population of GSCs is maintained adjacent to the distal tip cells (DTC), with entry into meiosis and maturation of gametes again occurring at subsequently more proximal locations along the gonad arms. In contrast, no such sustained polarity is observed in the *O. dioica* ovary. The polyploid “inner trinucleate cell” (ITC), which may have a signaling role [13], is initially juxtaposed to the gonad cavity which abuts the intestine but subsequently becomes surrounded by the unicellular, multinucleate, ovarian coenocyst. Entry into meiosis at day 3 is essentially synchronous throughout the entire ovary and after meiotic entry, there is no population of GSCs maintained in proximity to the ITC or elsewhere in the ovary [14], [15]. We examined to what extent this different reproductive mode and organization of oogenesis impacts conserved signaling pathways, identified in *Drosophila* and C. *elegans,* linking GA and reproductive state under nutrient restricted conditions.

### *O. dioica* Growth Arrest occurs Immediately Prior to Meiotic Entry in the Female Germline

In *O. dioica,* GA was only observed to appear at the midpoint of the life cycle, on day 3, immediately prior to the time at which females would normally enter meiosis. Below a minimum threshold of nutrition, animals do not enter GA, instead dying before they are able to spawn [46]. Animals could remain arrested at this day 3 morphological state for at least three times the normal life cycle, and, upon release from GA resumed maturation and spawning in a normal time frame with a normal reproductive output. On the other hand, if animals were exposed to crowded nutrient-limited conditions after meiotic entry in the female germline, they were unable to arrest. Instead they matured and spawned with normal lifespan, but substantially reduced reproductive output.

### TOR and MAPK Signaling in *O. dioica* Growth Arrest

Upon entry into GA, somatic endocycle progression ceased immediately whereas somatic mitotic intestinal cells and endomitotic germline syncytial cycles were not initially affected, but gradually declined in their progression over the duration of the nutrient-limited period. Upon release from GA, it was also the mitotic somatic and germline cells that initially re-entered the cell cycle, with polyploid cells resuming their endocycles afterwards. In *Drosophila*, amino acid withdrawal also immediately arrests endocycling cells but not mitotic cells [7]. Furthermore, loss of TOR function reduces cytoplasmic volume in endocycling tissues whereas mitotic cells grow normally in *Drosophila* and *C. elegans* [48], [49], [58]. In *O. dioica*, under normal nutritive conditions but with direct inhibition of TOR activity by CCI-779, similar arrest results were obtained with respect to these different cell cycle variants. These findings agree with those in other organisms where attenuated TOR signaling has been associated with GA. Inactivation of *CeTOR* or TOR signaling component *DAF 15* (raptor) induces larval arrest and increases lifespan in *C. elegans* [48], [49]. Similarly *tor* homozygous mutants produce arrested larvae in *Drosophila* [50].

Notably, inhibition of TOR activity alone prevented meiotic entry in *O. dioica*, thus also blocking the asymmetric nuclear germline differentiation into prophase I or endocycling nurse nuclei that would normally occur [14], [15]. TSC1/2 a negative regulator of TOR signaling is known to prevent germ cell differentiation in *Drosophila* [59] and TOR inhibition is proposed to suppress the translation of meiotic onset specific genes in *Saccharomyces* [60] In the GA state in *O. dioica,* we found that inhibition of the MAPK ERK1/2 or p38 signaling pathways resulted in significantly decreased survival, consistent with role of these evolutionarily conserved pathways in stress survival in other organisms. Under crowded nutrient-limited GA conditions, the active forms of both *O. dioica* MAPK ERK1/2 (nuclear) and p38 (cytoplasmic) were increased in the endomitotic germline as was their common downstream effector, MSK1. On the other hand, under normal nutritive conditions, inhibition of TOR signaling by CCI-779 activated p38 and MSK1 but not ERK, indicating a mechanistic link between the absence of TOR signaling and activation of p38–MSK1 signaling, but not the ERK pathway in *O. dioica*, under these conditions. This suggests that absence of TOR signaling during GA triggers the downstream activation of survival p38–MSK1 signaling in the endomitotic germline whereas parallel activation of ERK1/2 appears independent of TOR signaling. ERK1/2 and p38 signaling play roles in increasing longevity in *C. elegans* [28], [61] and p38-dependent MSK1 activity promotes survival in mammalian cells [41]. We conclude that the activation of ERK1/2, p38 and MSK1, promotes survival and longevity in *O. dioica* during nutrient-limited conditions where TOR activity is reduced.

### Regulators of Cell Cycle Transitions under Conditions Leading to Growth Arrest

Our results indicate that the rapid arrest of *O. dioica* somatic endocycling cells under GA conditions where TOR signaling is reduced, or under direct inhibition of TOR signaling alone, acts at the level of a G1-S-like transition in these G-S cell cycle variants. The mRNA levels of the G1-S regulator *O. dioica cyclin Dd* were unaffected in arrested endocycling cells, whereas a drastic decrease in the level of Cyclin Dd protein was observed. Cyclin D, the translation of which is regulated by a 5′ TOP dependent mechanism, is a well-known target of TOR signaling, and Cyclin Dd thus acts as a growth sensor in somatic endocycling cells similar to other organisms. Another major G1-S regulator from the Cip/Kip family, Cyclin dependent kinase inhibitor (CKI) has been reported to inhibit the cell cycle under starvation and TOR inhibition [62]–[66]. CKI inhibits the CDK/2-cyclin E complex [65], [67] and PCNA in the nucleus [68] thereby arresting the cell cycle in a G0 state. TOR signaling also negatively controls the stability of p27, a Cyclin Dependent Kinase Inhibitor, in mammalian cells [38], [53]. In *O. dioica*, during TOR inhibition, we found both mRNA and protein levels of the Cyclin Dependent Kinase Inhibitor a (CKIa) to be upregulated, with CKIa localized to the nucleus of arrested endocycling cells. Interestingly, daf-16, a FOXO transcription factor promotes the transcription of CKI-1 which induces cell cycle arrest in L1 arrested C. *elegans* [64]. FOXO transcription factors also regulate the transcription of p27 in mammalian cells [69]. Consistent with these findings, we found the ortholog of FOXO/daf-16 was up regulated in GA *O. dioica* (Danks et al., unpublished data) suggesting a conserved regulatory circuit in the up-regulation of CKI transcripts.

The E2F family of transcription factors also play important roles in the progression of mitotic and endoreduplicative cell cycles. Under standard culture conditions, *O. dioica* E2F1 was constitutively present in both G-and S-phases of somatic endocycling cells but was restricted to G-phase in germ line mitotic nuclei. This *O. dioica* endocycle result contrasts observations in *Drosophila* salivary gland cells where E2F1 is expressed in G-phase but degrades during S-phase in endocycling cells [55]. Proteolytic, S-phase, destruction of *Drosophila* E2F1 depends on an E2F1 PIP box motif that binds PCNA and mediates interaction with the CRL4 ubiquitin ligase [70]. Interestingly, *O. dioica* E2F1 lacks the PIP box motif (**Fig. S6**), suggesting altered details in the regulatory mechanisms controlling these somatic endocycle types in the two species. The importance, however, of E2F1 for somatic endocycle progression in *O. dioica* was demonstrated during GA. Under these conditions, E2F1 mRNA levels were not affected but protein levels became almost undetectable and somatic endocycles ceased. Thus, the translation of E2F1 was responsive to nutrient availability in a TOR-dependent manner, as seen in *Drosophila* [55].

### Conclusions

In the iteroparous reproductive models, *C. elegans* and *Drosophila*, reduced rates of oogenesis can occur post-meiotic entry. When nutrition is limiting, and TOR signaling inhibited, the GSCs of these species do reduce in number but a pool of active stem cells is maintained, in contrast to many of the post-meiotic cells which are destroyed and harvested for the energy reserves they represent. In *C. elegans*, oogenesis proceeds, though at a rate approximately 80-fold slower than in animals with adequate nutrition [5]. This reduced sensitivity of GSCs to the nutrition-TOR signaling-G1/S transition axis may be related to the observation that the GSCs of these species possess a very short G1-phase with most cycle regulation mediated by Cyclin E which persists in G2 [71], [72]. In distinct contrast, nutrient-limited GA only occurred prior to meiotic entry in the semelparous *O. dioica*. In this case, the GSC endomitotic nuclei were also resistant to proliferative arrest in response to nutritional restriction or direct TOR inhibition, though the oscillation of E2F1 levels is suggestive of some G1/S regulation in this stem line. Thus, the link between reproductive status and lifespan extension in response to nutrient-limited conditions is interpreted in a significantly different manner in these iteroparous versus semelparous models. In the latter case, meiotic entry is a definitive signal that lifespan extension can no longer occur, whereas in the former, meiotic entry is a not a unique chronological event, and can be largely erased during lifespan extension in response to nutrient stress, and reactivated from a pool of maintained GSCs when conditions improve.

## Supporting Information

Figure S1 ***Oikopleura dioica*** **maturation.** At standard culture conditions, the day 3 mitotic gonad is transparent and germ line nuclei do not fully occupy the gonad cavity. This is also the state of the gonad at day 3 in animals cultured under dense conditions. At day 6, maturation of the ovary is easily apparent and oocytes are apparent [43] in animals cultured under standard conditions, whereas animals cultured under dense conditions exhibit a day 6 gonad morphology which remains very similar to that normally observed at day 3, with no evidence of maturation.Arrows indicate the gonad. Scale bars = 200 μm.
(TIF)

Figure S2 **DNA replication throughout the** ***Oikopleura dioica*** **life cycle.** Incorporation of IdU (S phase marker) in *O. dioica* somatic endocycling and germline nuclei from day 2 to 6 when cultured at standard densities. Upon reaching maturity (early day 6), somatic endocycles ceased, whereas the germline nuclei continued to incorporate IdU. Scale bars = 50 μm.
(TIF)

Figure S3 **DNA replication in mitotic intestinal cells during growth arrest (GA) and recovery from growth arrest.** A) *O. dioica* cultured under standard conditions in the presence of DMSO, in the presence of 7.5 μM TOR inhibitor CCI-779 (Rapamycin analog) for 24 h, in the absence of food for 24 h (starvation) or, at high densities (Dense), showed that the mitotically proliferating intestinal cells do not undergo cell cycle arrest immediately upon GA and continue to incorporate IdU. DNA replication ceased in the intestine after 12 days of GA. Scale bars = 50 μm. B) Experimental design: GA *O. dioica* were either maintained under GA or released into standard density culture conditions. After 2 or 3 h, they were exposed to sequential (1 h each) pulses of CldU and IdU. C) S-phase was restored in mitotic intestinal cells 1 h after mitotic germline nuclei and 1 h prior to that observed in somatic endocycling cells upon release from GA (compare with **Fig. 2**). Scale bars = 10 μm.
(TIF)

Figure S4 **Multiple sequence alignment of Cip/Kip sequences with** ***O. dioica*** **CKIa.** *O. dioica* CKIa was aligned with members of the Cip/Kip family from other species using MUSCLE [73]. Dark shading indicates identical conserved amino acid positions and grey shading represents similar amino acids. Cyclin and Cyclin-dependent kinase (CDK) Cip/Kip binding regions are indicated by double-headed arrows. Cyclin binding residues RXLF (red box) and the tyrosine residue (black circle), a phosphorylation site critical in the formation of non-inhibitory, active complexes with Cyclin D:CDK4/6 [74] are indicated. *Dm*dap, *Drosophila melanogaster* dacapo; *Mm*p27, *Mus musculus* p27; *Xl*Xic3, *Xenopus laevis Xic3*; *Od*CKIa, *Oikopleura dioica* CKIa; *Ce*CKI-1 *Caenorhabditis elegans* CKI1.
(TIF)

Figure S5 **Transcription profiles of G- to S-phase transition regulators during entry into growth arrest in** ***Oikopleura dioica*** **.** Quantitative real time PCR was performed using RNA isolated from *O. dioica* cultured under standard and dense conditions at day 2, 3, and 4. A) There was no change in the relative expression of *Cyclin Dd* transcripts in animals grown under dense culture conditions compared to the control standard culture animals. B) The relative transcript levels of Cyclin-dependent Kinase Inhibitor a (CKIa), did not change during culture at higher densities on day 2 and 3. However, *CKIa* expression was up-regulated (*p<0.05) at day 4 in culture at higher densities compared to animals cultured under standard conditions. C) The relative expression of the canonical transcription factor E2F1 was not affected in animals grown under dense culture conditions. Error bars indicate standard error.
(TIF)

Figure S6 **Multiple sequence alignment of E2F1 orthologs.** *O. dioica* E2F1 was aligned with E2F1 from other species using MUSCLE [73]. Dark shading indicates identical conserved amino acid positions and grey shading represents similar amino acids. The DNA binding domain (red box) [75] with the RRIYD DNA binding motif, the dimerization domain with DP proteins (blue box) and Rb binding groove (brown box) [76] are indicated. The PIP motif (green box) [70] is present only in *Drosophila*. Absence of the PIP motif in *O. dioica* E2F1 was verified using ELM (http://elm.eu.org). *Ce*ELF1, *Caenorhabditis elegans* ELF1; *Dm*E2F1, *Drosophila melanogaster* E2F1; *Od*E2F1, *Oikopleura dioica* E2F1; *Xl*E2F1, *Xenopus laevis* E2F1; *Mm*E2F1, *Mus musculus* E2F1.
(TIF)

Table S1 **Primers for qPCR.**
(PDF)
