# Supplementary figures and images for: Lifespan Extension in a Semelparous Chordate Occurs via Developmental Growth Arrest Just Prior to Meiotic Entry
Source: PLoS One. 2014 Apr 2;9(4):e93787. doi: 10.1371/journal.pone.0093787 (PMC3973624; doi:10.1371/journal.pone.0093787)

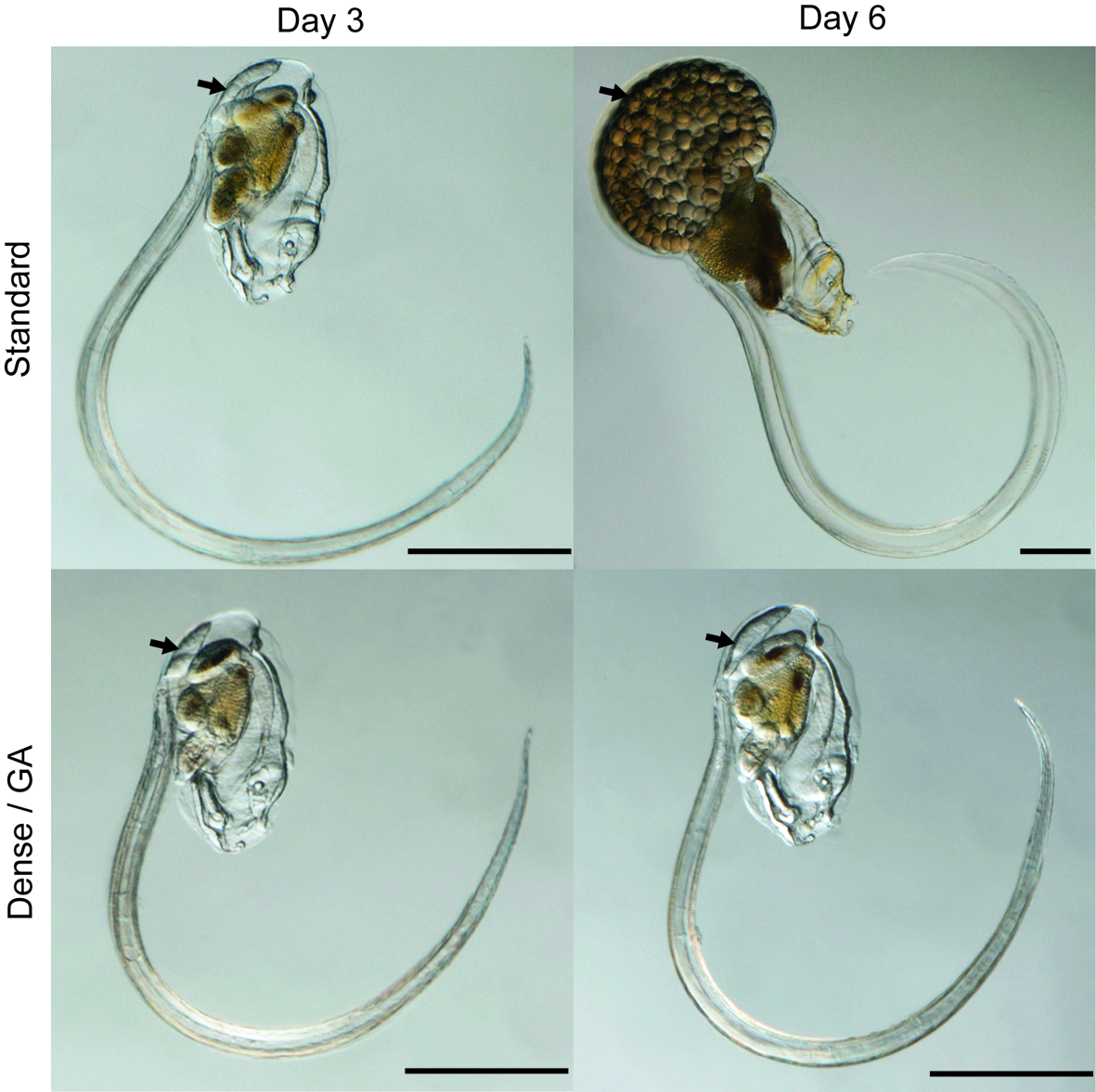

Supplement: Figure S1 — Oikopleura dioica maturation. At standard culture conditions, the day 3 mitotic gonad is transparent and germ line nuclei do not fully occupy the gonad cavity. This is also the state of the gonad at day 3 in animals cultured under dense conditions. At day 6, maturation of the ovary is easily apparent and oocytes are apparent [43] in animals cultured under standard conditions, whereas animals cultured under dense conditions exhibit a day 6 gonad morphology which remains very similar to that normally observed at day 3, with no evidence of maturation.Arrows indicate the gonad. Scale bars = 200 μm. (TIF) [file pone.0093787.s001.tif]

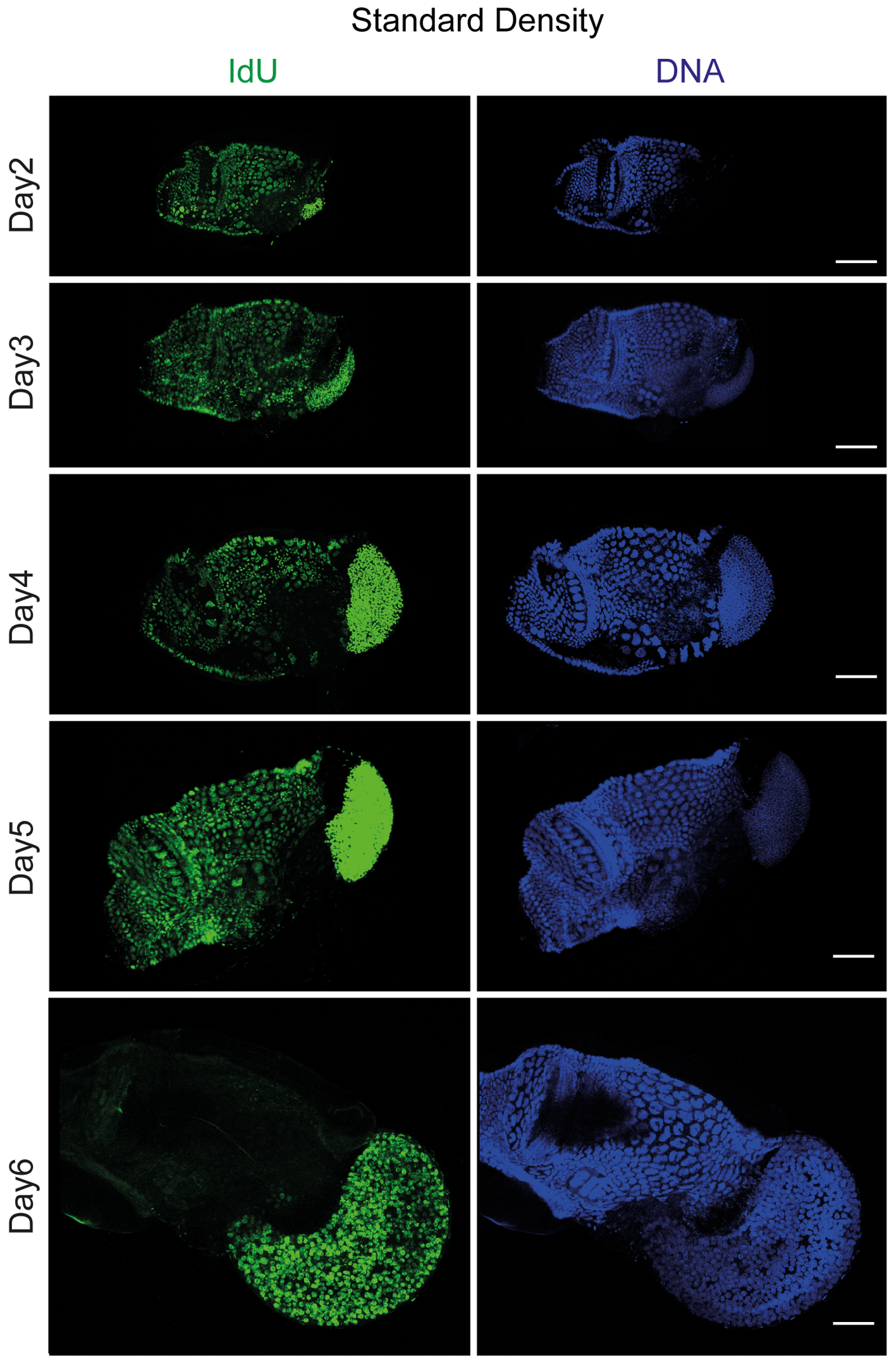

Supplement: Figure S2 — DNA replication throughout the Oikopleura dioica life cycle. Incorporation of IdU (S phase marker) in O. dioica somatic endocycling and germline nuclei from day 2 to 6 when cultured at standard densities. Upon reaching maturity (early day 6), somatic endocycles ceased, whereas the germline nuclei continued to incorporate IdU. Scale bars = 50 μm. (TIF) [file pone.0093787.s002.tif]

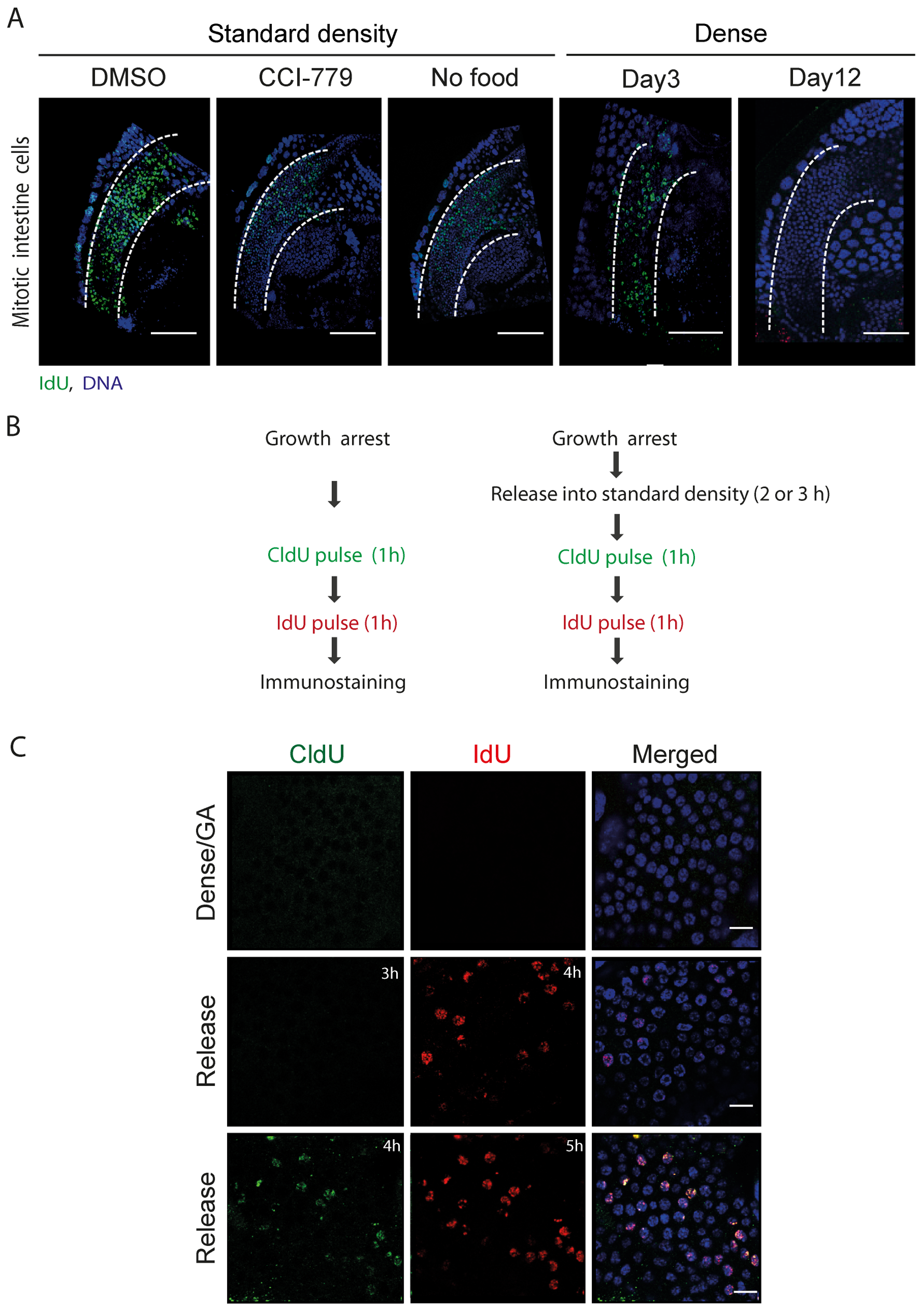

Supplement: Figure S3 — DNA replication in mitotic intestinal cells during growth arrest (GA) and recovery from growth arrest. A) O. dioica cultured under standard conditions in the presence of DMSO, in the presence of 7.5 μM TOR inhibitor CCI-779 (Rapamycin analog) for 24 h, in the absence of food for 24 h (starvation) or, at high densities (Dense), showed that the mitotically proliferating intestinal cells do not undergo cell cycle arrest immediately upon GA and continue to incorporate IdU. DNA replication ceased in the intestine after 12 days of GA. Scale bars = 50 μm. B) Experimental design: GA O. dioica were either maintained under GA or released into standard density culture conditions. After 2 or 3 h, they were exposed to sequential (1 h each) pulses of CldU and IdU. C) S-phase was restored in mitotic intestinal cells 1 h after mitotic germline nuclei and 1 h prior to that observed in somatic endocycling cells upon release from GA (compare with Fig. 2 ). Scale bars = 10 μm. (TIF) [file pone.0093787.s003.tif]

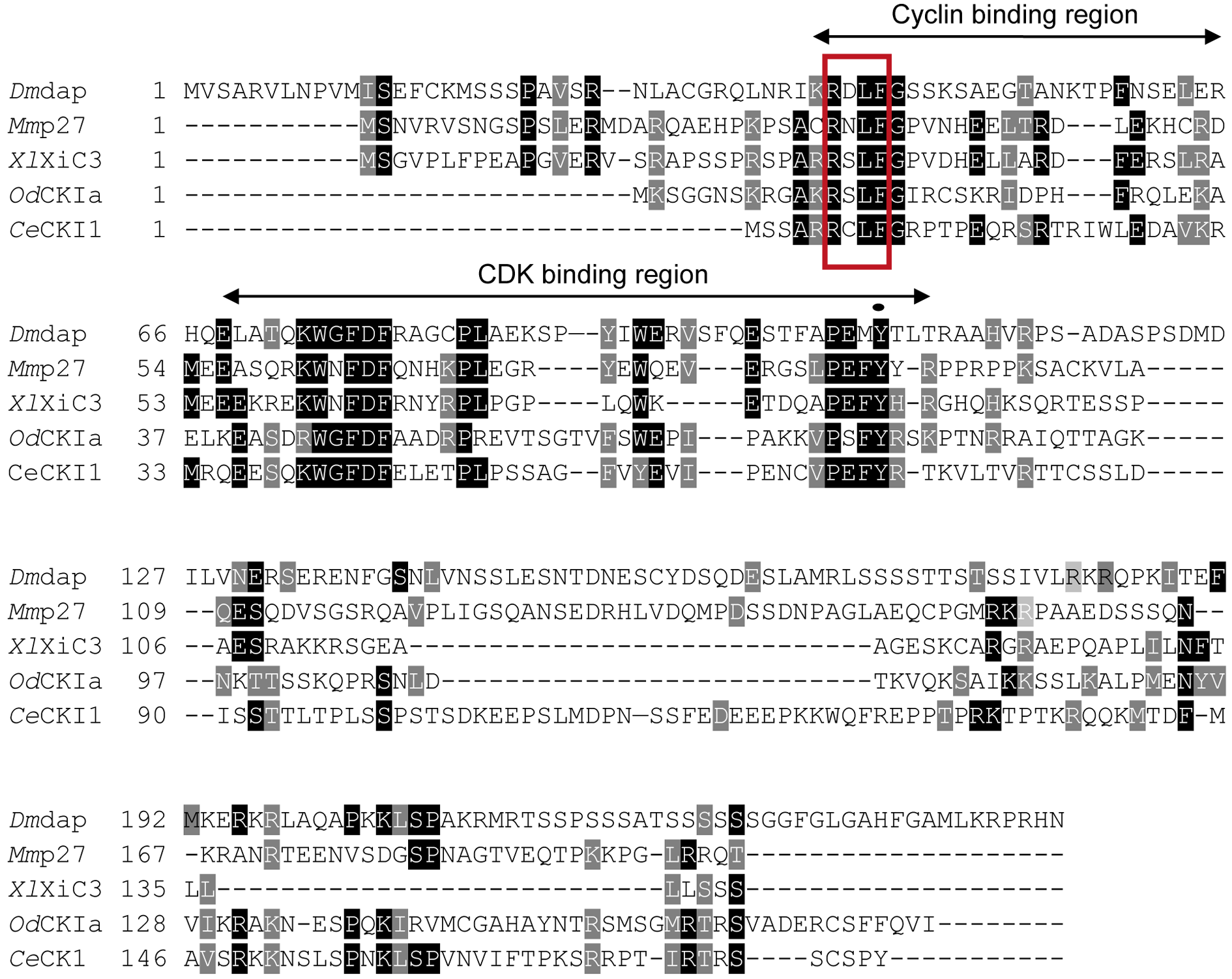

Supplement: Figure S4 — Multiple sequence alignment of Cip/Kip sequences with O. dioica CKIa. O. dioica CKIa was aligned with members of the Cip/Kip family from other species using MUSCLE [73]. Dark shading indicates identical conserved amino acid positions and grey shading represents similar amino acids. Cyclin and Cyclin-dependent kinase (CDK) Cip/Kip binding regions are indicated by double-headed arrows. Cyclin binding residues RXLF (red box) and the tyrosine residue (black circle), a phosphorylation site critical in the formation of non-inhibitory, active complexes with Cyclin D:CDK4/6 [74] are indicated. Dmdap, Drosophila melanogaster dacapo; Mmp27, Mus musculus p27; XlXic3, Xenopus laevis Xic3; OdCKIa, Oikopleura dioica CKIa; CeCKI-1 Caenorhabditis elegans CKI1. (TIF) [file pone.0093787.s004.tif]

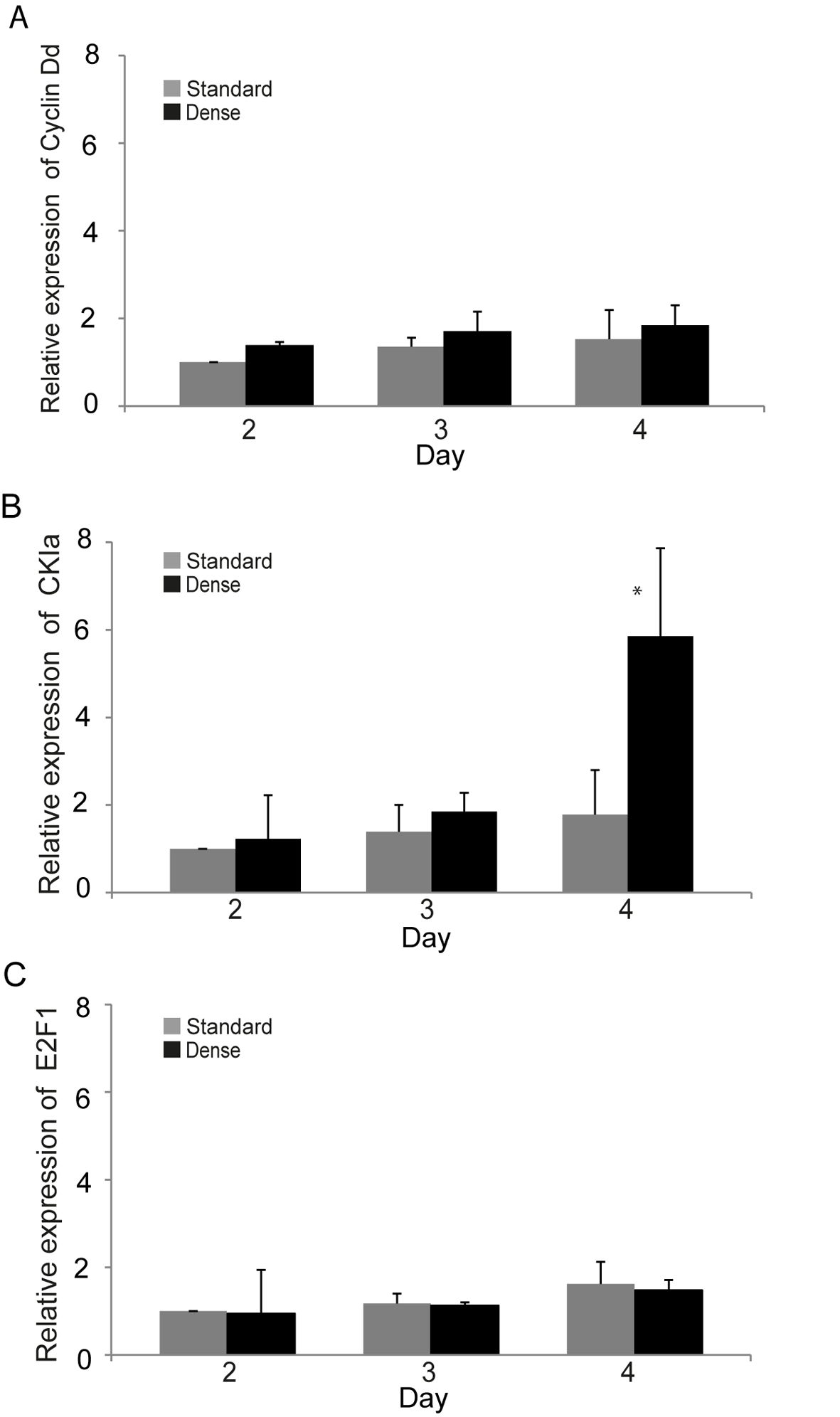

Supplement: Figure S5 — Transcription profiles of G- to S-phase transition regulators during entry into growth arrest in Oikopleura dioica . Quantitative real time PCR was performed using RNA isolated from O. dioica cultured under standard and dense conditions at day 2, 3, and 4. A) There was no change in the relative expression of Cyclin Dd transcripts in animals grown under dense culture conditions compared to the control standard culture animals. B) The relative transcript levels of Cyclin-dependent Kinase Inhibitor a (CKIa), did not change during culture at higher densities on day 2 and 3. However, CKIa expression was up-regulated (*p<0.05) at day 4 in culture at higher densities compared to animals cultured under standard conditions. C) The relative expression of the canonical transcription factor E2F1 was not affected in animals grown under dense culture conditions. Error bars indicate standard error. (TIF) [file pone.0093787.s005.tif]

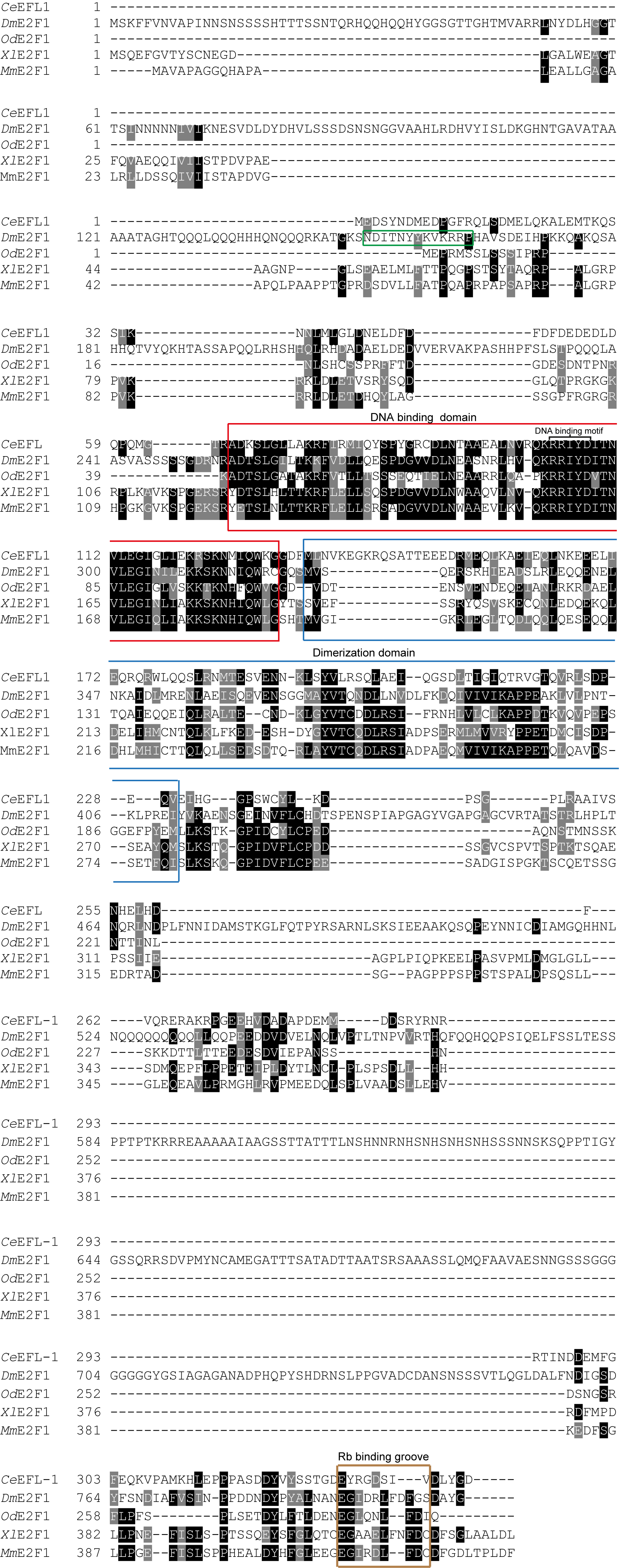

Supplement: Figure S6 — Multiple sequence alignment of E2F1 orthologs. O. dioica E2F1 was aligned with E2F1 from other species using MUSCLE [73]. Dark shading indicates identical conserved amino acid positions and grey shading represents similar amino acids. The DNA binding domain (red box) [75] with the RRIYD DNA binding motif, the dimerization domain with DP proteins (blue box) and Rb binding groove (brown box) [76] are indicated. The PIP motif (green box) [70] is present only in Drosophila. Absence of the PIP motif in O. dioica E2F1 was verified using ELM (http://elm.eu.org). CeELF1, Caenorhabditis elegans ELF1; DmE2F1, Drosophila melanogaster E2F1; OdE2F1, Oikopleura dioica E2F1; XlE2F1, Xenopus laevis E2F1; MmE2F1, Mus musculus E2F1. (TIF) [file pone.0093787.s006.tif]
