# Supplementary material for: Lifespan Extension in a Semelparous Chordate Occurs via Developmental Growth Arrest Just Prior to Meiotic Entry
Source: PLoS One. 2014 Apr 2;9(4):e93787. doi: 10.1371/journal.pone.0093787 (PMC3973624; doi:10.1371/journal.pone.0093787)
Supplement: Table S1 — Primers for qPCR. (PDF) [file pone.0093787.s007.pdf]

Additional File 6: Table S1. Subramaniam et al.

Table S1. Primers for qPCR.

Target	Primer	Sequence (5′ to 3′)
Cyclin Dd	CCQ87F	TCCTGTCTATATCTGGCCGCGAAA
	CCQ88R	GGGCTTGGCAGAAGAGAAGAATGA
CKIa	CCQ141F	GTCGTCTTTGAAAGCGTTGCCGAT
	CCQ142R	ACATCGAGCGAGTGTTGTACGCAT
E2F1	CCQ260F	GCGGAACTCACACAAGCGATTGAA
	CCQ261R	AAGATTGAACGCAAGTCGTCGCAG
EF1β	CCQ43F	AGGTCATCCCTGAACTTAACGGCA
	CCQ44R	GGCAGATTTGATGGCAGCGTTGAT
